# Bioinformatic and functional characterization of cyclic-di-GMP metabolic proteins in *Vibrio alginolyticus* unveils key diguanylate cyclases controlling multiple biofilm-associated phenotypes

**DOI:** 10.3389/fmicb.2023.1258415

**Published:** 2023-09-21

**Authors:** Xiao-Xiao Gong, Yan-Hua Zeng, Hai-Min Chen, Na Zhang, Yue Han, Hao Long, Zhen-Yu Xie

**Affiliations:** ^1^State Key Laboratory of Marine Resource Utilization in the South China Sea, Hainan University, Haikou, Hainan, China; ^2^Hainan Provincial Key Laboratory for Tropical Hydrobiology and Biotechnology, Hainan University, Haikou, Hainan, China; ^3^College of Marine Sciences, Hainan University, Haikou, Hainan, China

**Keywords:** *Vibrio alginolyticus*, c-di-GMP, rugose, biofilm, extracellular polysaccharide

## Abstract

The biofilm lifestyle is critical for bacterial survival and proliferation in the fluctuating marine environment. Cyclic diguanylate (c-di-GMP) is a key second messenger during bacterial adaptation to various environmental signals, which has been identified as a master regulator of biofilm formation. However, little is known about whether and how c-di-GMP signaling regulates biofilm formation in *Vibrio alginolyticus*, a globally dominant marine pathogen. Here, a large set of 63 proteins were predicted to participate in c-di-GMP metabolism (biosynthesis or degradation) in a pathogenic *V. alginolyticus* strain HN08155. Guided by protein homology, conserved domains and gene context information, a representative subset of 22 c-di-GMP metabolic proteins were selected to determine which ones affect biofilm-associated phenotypes. By comparing phenotypic differences between the wild-type and mutants or overexpression strains, we found that 22 c-di-GMP metabolic proteins can separately regulate different phenotypic outputs in *V. alginolyticus*. The results indicated that overexpression of four c-di-GMP metabolic proteins, including VA0356, VA1591 (CdgM), VA4033 (DgcB) and VA0088, strongly enhanced rugose colony morphotypes and strengthened Congo Red (CR) binding capacity, both of which are indicators of biofilm matrix overproduction. Furthermore, rugose enhanced colonies were accompanied by increased transcript levels of extracellular polysaccharide (EPS) biosynthesis genes and decreased expression of flagellar synthesis genes compared to smooth colonies (WTpBAD control), as demonstrated by overexpression strains WTp4033 and ∆*VA4033*p4033. Overall, the high abundance of c-di-GMP metabolic proteins in *V. alginolyticus* suggests that c-di-GMP signaling and regulatory system could play a key role in its response and adaptation to the ever-changing marine environment. This work provides a robust foundation for the study of the molecular mechanisms of c-di-GMP in the biofilm formation of *V. alginolyticus.*

## Introduction

*Vibrio alginolyticus* is one of three most abundant *Vibrio* species found in marine and estuarine environments worldwide, which has caused frequent outbreaks of high-mortality vibriosis in aquatic animals (e.g., fish, shrimp and shellfish) and significant economic losses to the global aquaculture industry (Xie et al., 2005; Jacobs Slifka et al., 2017; Ibangha et al., 2023; Yu et al., 2023). The transition from a planktonic to a biofilm lifestyle is key to the survival and pathogenicity of *Vibrio* spp. (Baker-Austin et al., 2018). Bacterial cells encased in biofilms exhibit a range of emergent properties that are quite different from free-living cells, such as enhanced social cooperation, more efficient resource capture and increased resistance to antimicrobials (Flemming et al., 2016). Therefore, biofilms provide such an important fitness advantage for *Vibrio*, enhancing environmental adaptations and enabling them to persist in different niches, especially within the host (Teschler et al., 2015).

Different stages of biofilm formation, including surface attachment, matrix production, biofilm maturation and dispersal, are regulated by sophisticated regulatory networks (Yildiz et al., 2001; Wang et al., 2014; Townsley and Yildiz, 2015). Evidence is mounting that the near-ubiquitous second messenger cyclic diguanylate (c-di-GMP) plays a central role in different stages of biofilm formation in a diverse group of microorganisms (Purcell et al., 2012; Romling et al., 2013; Valentini and Filloux, 2019; Ahmad et al., 2020). In the case of *Vibrio* spp., the best-studied example is *Vibrio cholerae*, the causative agent of the human disease cholera. It is now generally accepted that high levels of c-di-GMP promote extracellular polysaccharide (EPS) production and enhance biofilm formation of *V. cholerae*, while low levels of c-di-GMP increase cell motility and cause biofilm dispersal (Liu et al., 2010; Conner et al., 2017; Biswas et al., 2020; Wu et al., 2020). Recent evidence obtained from one of the dominant marine *Vibrio* spp., *Vibrio parahaemolyticus*, has also made it clear that high c-di-GMP favors biofilm formation (Kimbrough et al., 2020; Martinez-Mendez et al., 2021). In addition, interfering with c-di-GMP metabolism and signaling has been shown to be a promising way to control biofilm development (Liu et al., 2022; Kim et al., 2023). Therefore, modulation of the intracellular c-di-GMP pool is a key regulator of bacterial adaptation to sessile and motile lifestyles in response to complex environmental and internal cues.

The intracellular c-di-GMP pool is modulated by the competing activities of two classes of enzymes, diguanylate cyclases (DGCs) and phosphodiesterases (PDEs), which synthesize and degrade c-di-GMP, respectively. Functional DGCs typically contain a catalytically active GGDEF structural domain, while PDEs generally contain an EAL or HD-GYP active domain (Schirmer and Jenal, 2009; Romling et al., 2013). In most cases, bacterial genomes encode multiple DGCs and PDEs, however, their abundance varies among species or strains (Romling et al., 2013; Chou and Galperin, 2016). The number of genes encoding c-di-GMP metabolic proteins in *Vibrio* spp. is usually more than 50, e.g., the pathogens *V. cholerae*, *V. parahaemolyticus*, and *V. vulnificus* contain 62, 62, and 92 potential DGCs/PDEs, respectively (Shrestha et al., 2022). As recently reported, the number of c-di-GMP metabolic proteins in *Vibrio* spp. far exceeds that of *Escherichia coli* (Homma and Kojima, 2022), suggesting that the c-di-GMP signaling network in *Vibrio* is quite complex, which is crucial for them to sense environmental changes and initiate adaptive responses.

Notably, not all the DGCs/PDEs control the same c-di-GMP-dependent phenotypes, i.e., individual different DGCs/PDEs can generate distinct and specific output functions (Dahlstrom and O'Toole, 2017). In the case of *V. cholerae*, distinct c-di-GMP metabolic proteins contribute to different stages of biofilm progression (Massie et al., 2012; Zamorano-Sanchez et al., 2019). For example, among the 28 DGCs of *V. cholerae*, three specific DGCs, namely CdgA, CdgL and CdgO, are required for the flagellum-dependent biofilm regulatory (FDBR) response that enhances biofilm formation (Wu et al., 2020). In *Pseudomonas aeruginosa* PA14, the DGC SadC interacts with the type 4 pili (T4P) alignment complex protein PilO, and this interaction is an important regulatory hub for controlling early biofilm formation (Webster et al., 2021). In *E. coli*, the c-di-GMP metabolic proteins DgcE, DgcM, and PdeR specifically affect biofilm formation but are independent of effects on cellular c-di-GMP levels (Sarenko et al., 2017). Thus, bacteria can optimize phenotypic output to c-di-GMP levels via activation of specific c-di-GMP metabolic proteins as a response to specific environmental signals. As one of the most abundant *Vibrio* species in the global ocean, the ability to rapidly respond to environmental signals and transition to a biofilm lifestyle is critical for the survival of *V. alginolyticus* under adverse conditions (Yin et al., 2021, 2022). However, little attention has been paid to the c-di-GMP-mediated signal transduction system in *V. alginolyticus*, and it is unclear which specific c-di-GMP metabolic proteins play regulatory roles in the biofilm-associated phenotypes.

In this study, we first determined the number of c-di-GMP metabolic proteins encoded in the genome of *V. alginolyticus* HN08155, which we had previously isolated from diseased grouper and was highly virulent to *Litopenaeus vannamei* (Xie et al., 2020). A representative subset of c-di-GMP metabolic proteins was further selected and their individual functions in controlling biofilm-associated phenotypes were investigated. We constructed 44 derivative strains with each of the 22 DGC or PDE genes knocked out or overexpressed, and compared their colony morphology, biofilm formation, EPS production and swarming motility ability with the wild-type strain of *V. alginolyticus* HN08155. Overall, this study provided the first systematic bioinformatic and functional characterization of c-di-GMP metabolic proteins in *V. alginolyticus* and identified the specific DGCs required for biofilm formation in this dominant marine pathogen.

## Results

### Bioinformatic characterization of potential c-di-GMP metabolic proteins

A total of 63 proteins involved in c-di-GMP biosynthesis and degradation were identified in the genome of *V. alginolyticus* HN08155, of which 32 proteins contain only the GGDEF domain (hereinafter referred to as “GGDEF-only protein”), 11 proteins contain only the EAL domain (hereinafter “EAL-only protein”), 4 proteins contain only the HD-GYP domain (hereinafter “HD-GYP-only protein”), and 16 proteins contain both the GGDEF and EAL domains (hereinafter “hybrid protein”; Table 1).

**Table 1:** Structure prediction of 63 potential c-di-GMP metabolic proteins of *V. alginolyticus* HN08155 by the SMART algorithm and NCBI’s Conserved Domains Database.

Class	Protein	GGDEF domain	EAL /HD-GYP domain	I_S_	Domain architecture	Length (aa)
GGDEF-only proteins	VA1554	RxGGEEF	-	**√**	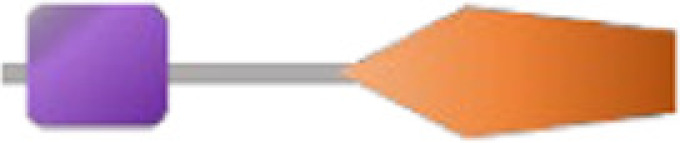	320
	VA4570	RxGGDEF	-	-	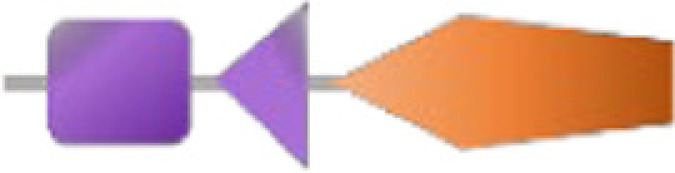	320
	VA0356	RxGGEEF	-	**√**	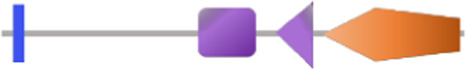	626
	VA0461	RxGGEEF	-	**√**	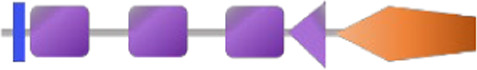	582
	VA4663	RxSGDEF	-	-	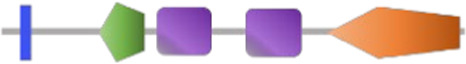	652
	VA2975	RxGGDEF			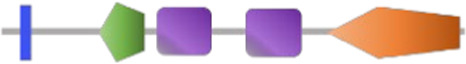	301
	VA0931	RxGGDEF	-	-	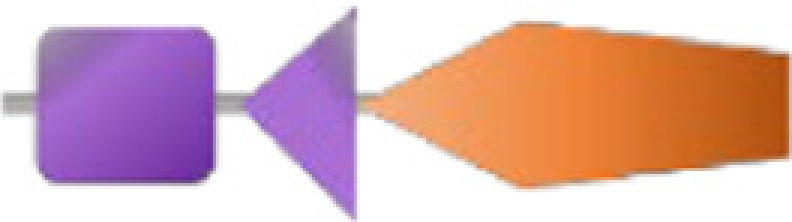	669
	VA4342	RxGGEEF	-	-	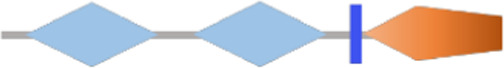	480
	VA4030	RxGGDEF	-	**√**	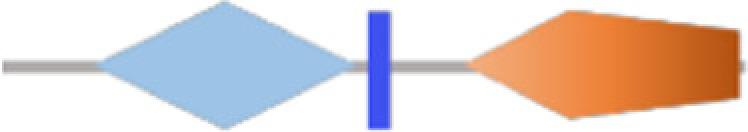	451
	VA1591 (*cdgM*)	RxGGDEF	-	-	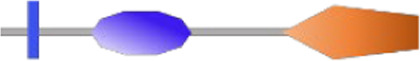	513
	VA4622 (*pleD*)	RxGGEEF	-	-	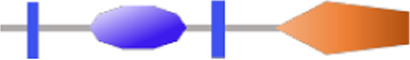	319
	VA3556	RxGGEEF	-	-	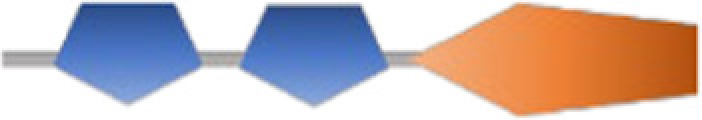	706
	VA1419	RxGGEEF	-	-	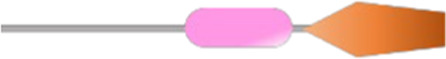	657
	VA4012	RxGGDEF	-	-	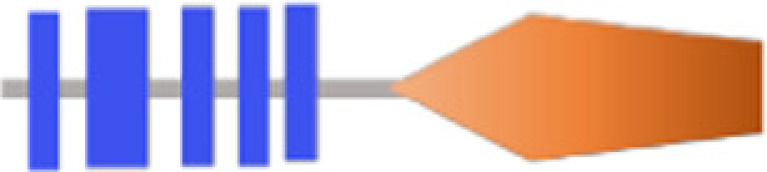	402
	VA4624	RxGGEEF	-	-	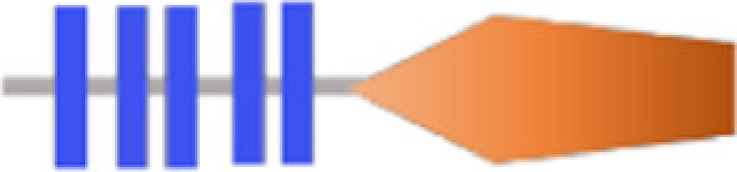	386
	VA3192	RxGGDEF	-	**√**	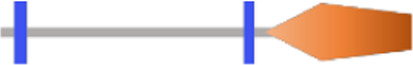	453
	VA1626	RxGGEEF	-	**√**	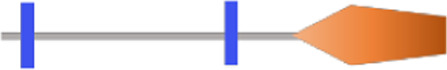	525
	VA2058 (*cdgK*)	RxGGEEF	-	**√**	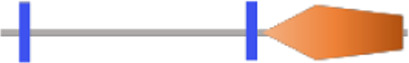	491
	VA2972	RxGGEEF	-	**√**	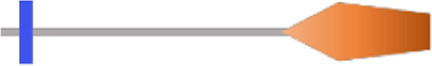	529
	VA1192	RxGGEEF	-	-	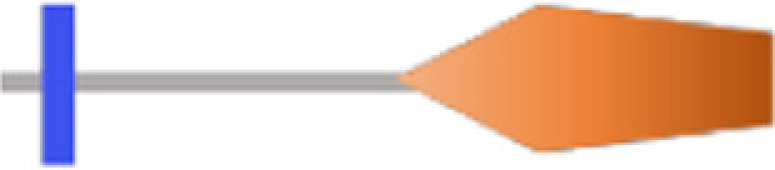	445
	VA4496	RxGGEEF	-	**√**	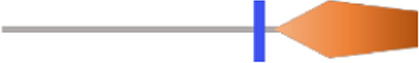	637
	VA3275	RxGGEEF	-	**√**	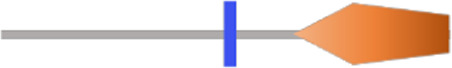	682
	VA3903 (*cdgA*)	RxGGDEF	-	-	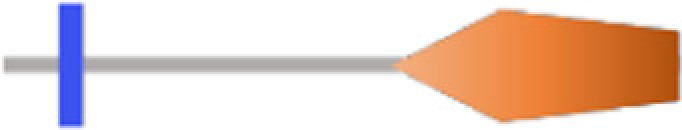	365
	VA3580	RxGGEEF	-	-	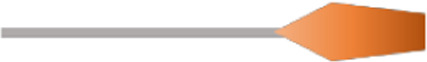	527
	VA0663	RxGGEEF	-	-	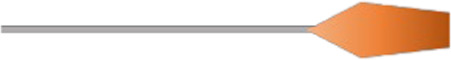	521
	VA4033 (*dgcB*)	RxGGEEF	-	-	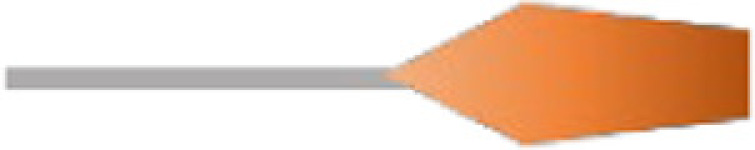	337
	VA4271	RxGGEEF	-	-	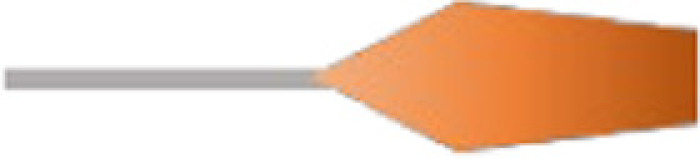	349
	VA2336	RxGGEEF	-	-	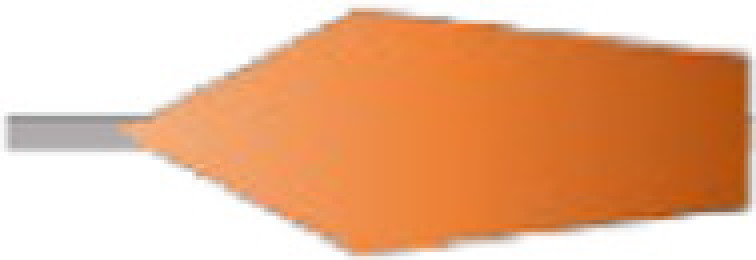	132
	VA3895	Rx**A**GDEF	-	-	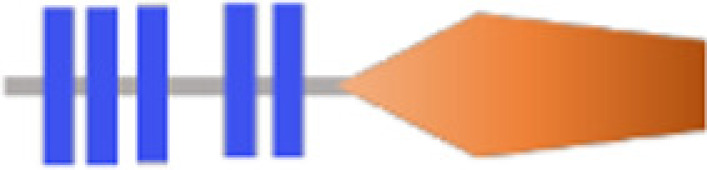	382
	VA1875	**E**x**TENAS**	-	-	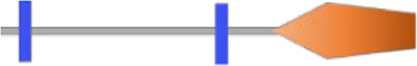	571
	VA2973	Rx**HS**D**G**F	-	-	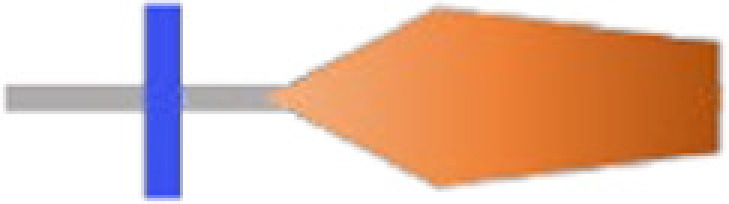	271
	VA3494	**Q**x**RLG**E**V**	-	-	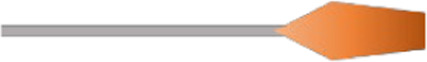	451
EAL-only proteins	VA1964	-	EAL	-	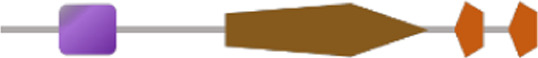	818
	VA3193	-	EAL	-	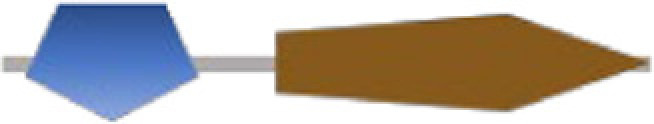	384
	VA4177	-	EAL	-	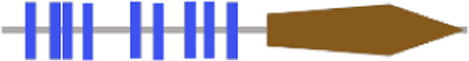	681
	VA1287	-	EAL	-	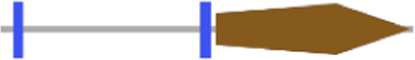	498
	VA4408	-	EAL	-	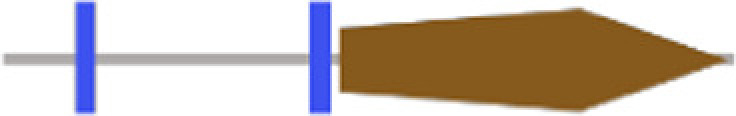	490
	VA1095	-	ELL	-	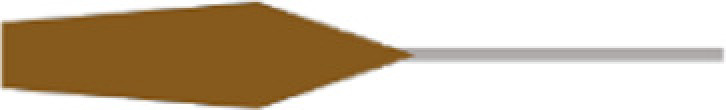	404
	VA2974	-	EVL	-	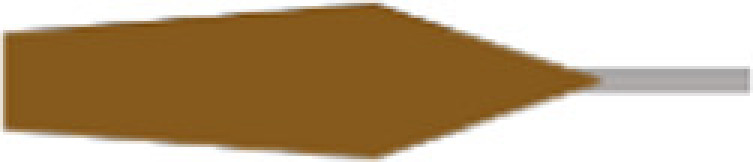	268
	VA3005 (*cdgJ*)	-	ELL	-	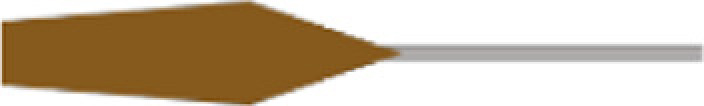	407
	VA3641	-	**HLT**	-	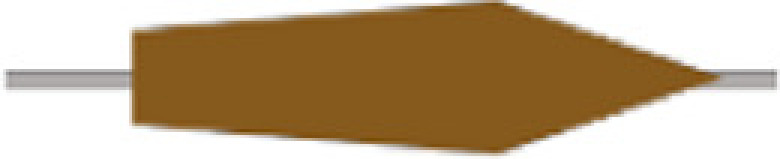	265
	VA4700	-	EAL	-	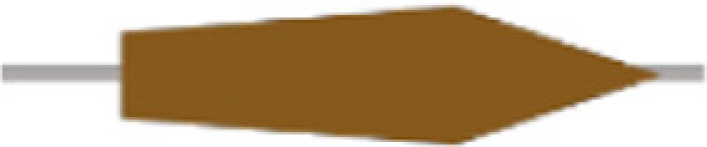	262
	VA4720	-	EAL	-	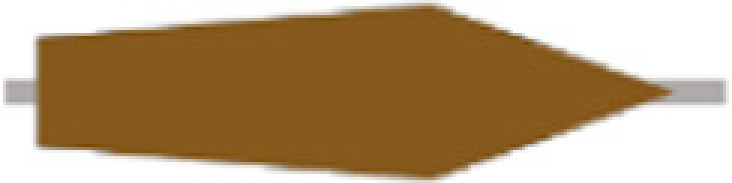	272
HD-GYP-only proteins	VA1608	-	HA-GYP	-	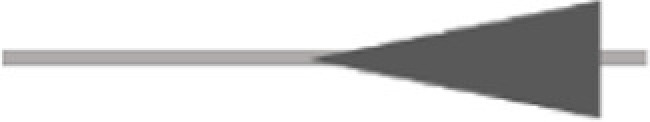	374
	VA1611	-	HD-GYP	-	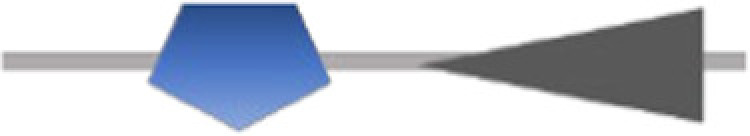	421
	VA1618	-	HD-GYP	-	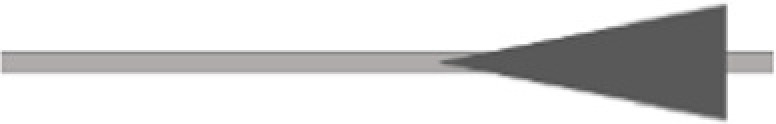	405
	VA2624	-	HD-GYP	-	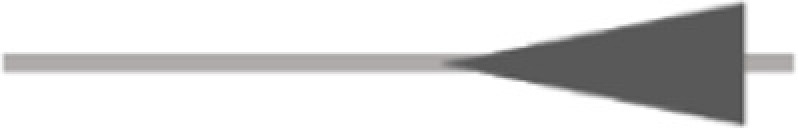	417
Hybrid proteins	VA0068	RxGGDEF	EIL	-	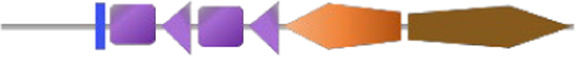	1,041
	VA0088	RxGGDEF	ETL	-	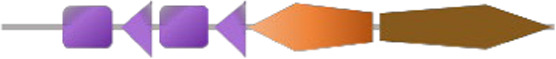	815
	VA2417 (*rocS*)	**H**xGGDEF	EAL	-	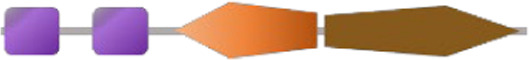	679
	VA3012	SxG**AG**E**W**	ECL	-	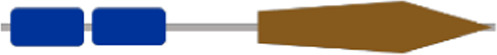	828
	VA3264	Rx**SEH**EF	EAL	-	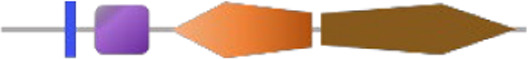	882
	VA1619	Rx**HE**D**D**F	ESL	-	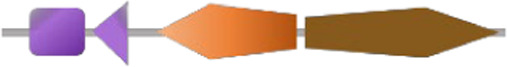	567
	VA0108	RxGGDEF	EAL	-	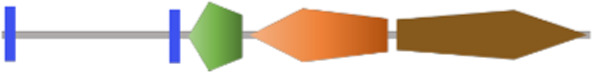	848
	VA1297	RxG**A**DEF	EAL	-	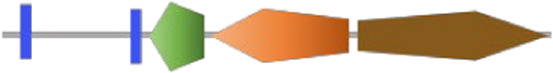	719
	VA3251 (*mbaA*)	Rx**S**GDEF	EVL	-	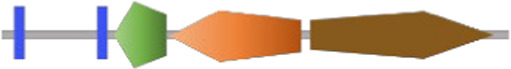	788
	VA3814 (*lapD*)	Rx**SS**DEF	EV**F**	-	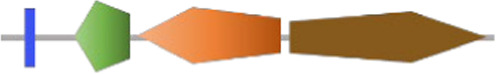	637
	VA4309	Rx**A**GDEF	EIL	-	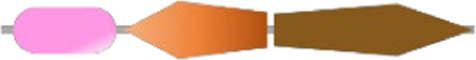	606
	VA4672 (*cdgC*)	Rx**SSNDL**	EAL	-	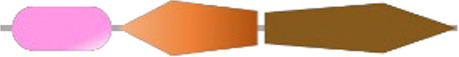	636
	VA3608	RxGGDEF	EAL	**√**	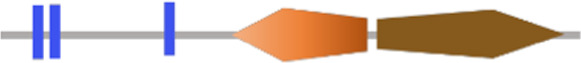	776
	VA4418	RxGGDEF	EA**V**	-	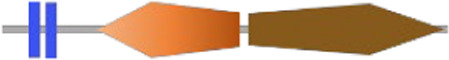	512
	VA2701 (*csrD*)	Rx**YDAD**F	EL**T**	-	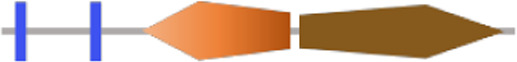	669
	VA3369	Rx**AS**DEF	EAL	-	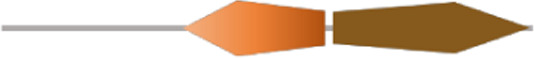	751

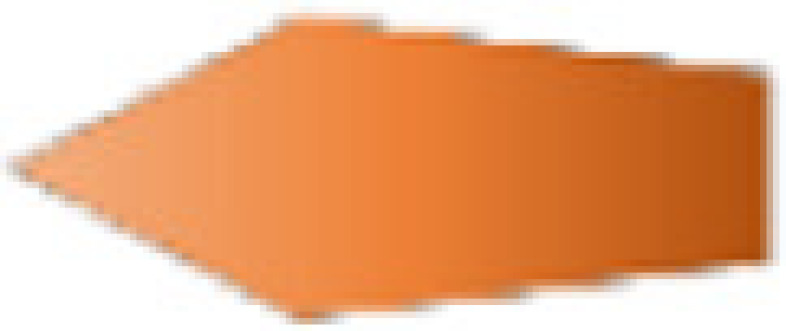
 GGDEF; 
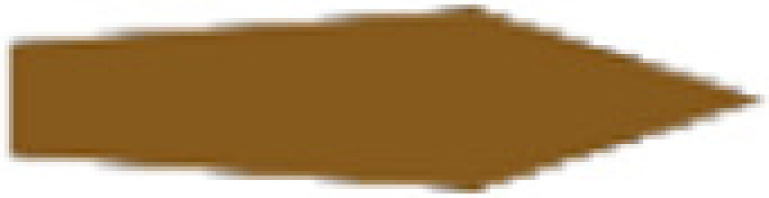
 EAL; 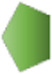 HAMP; 
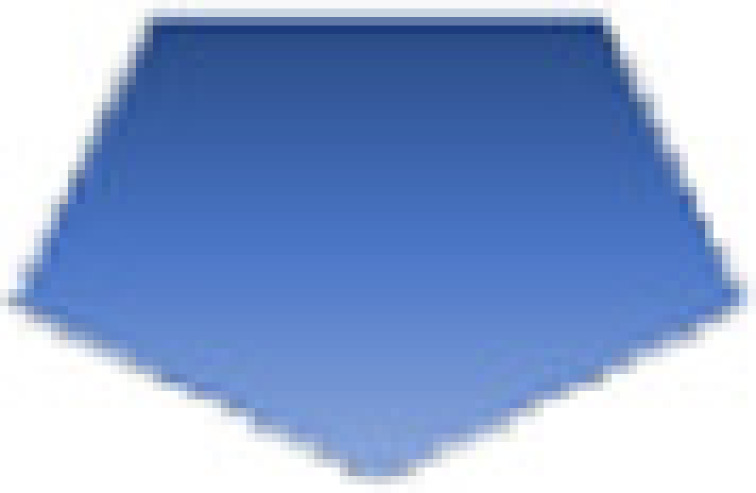
 REC; 
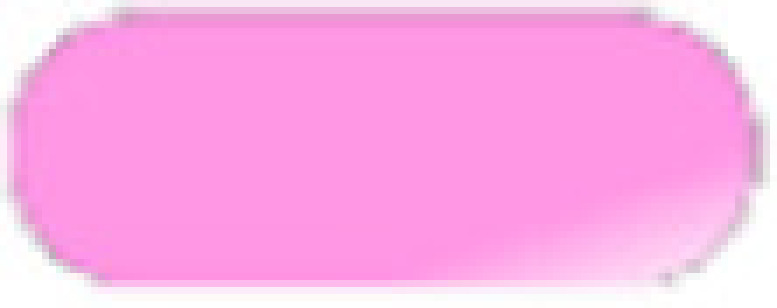
 GAF; 
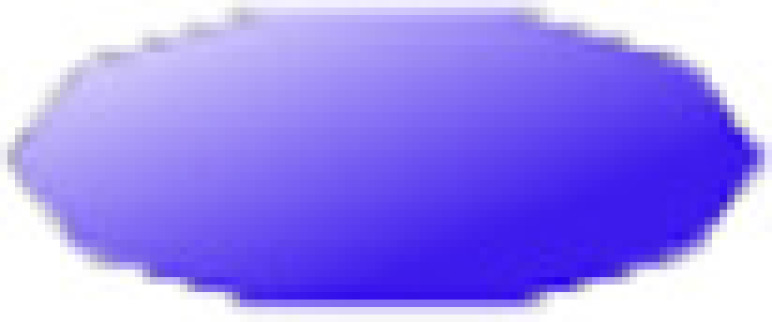
 CHASE; 
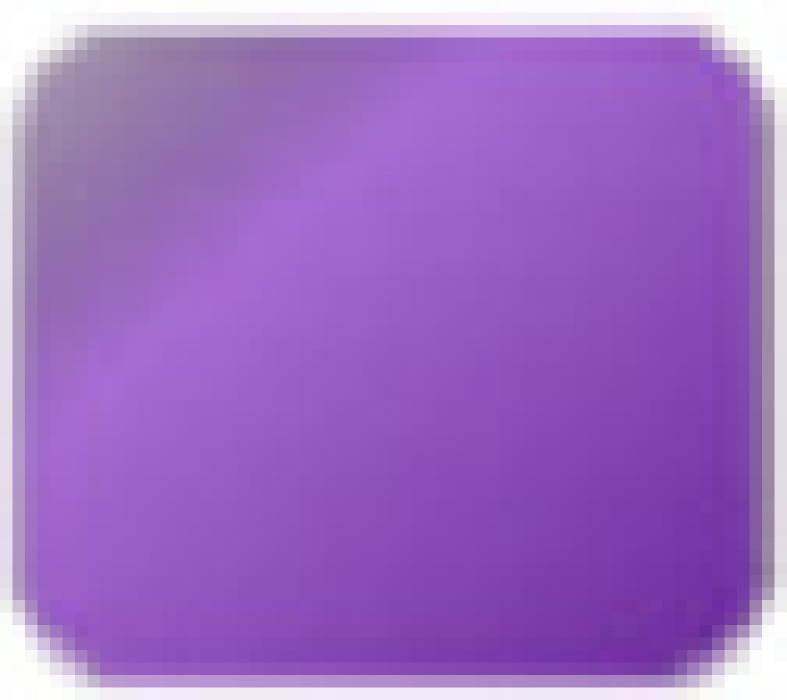
 PAS; 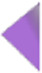 PAC; 
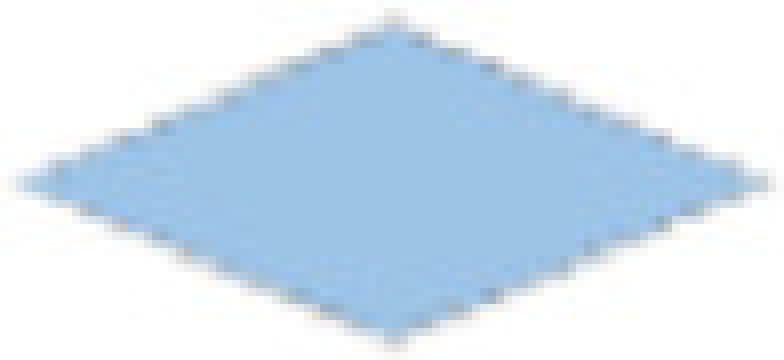
 PBP2; 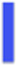 TM; 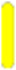 TPR; 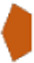 CBS; 
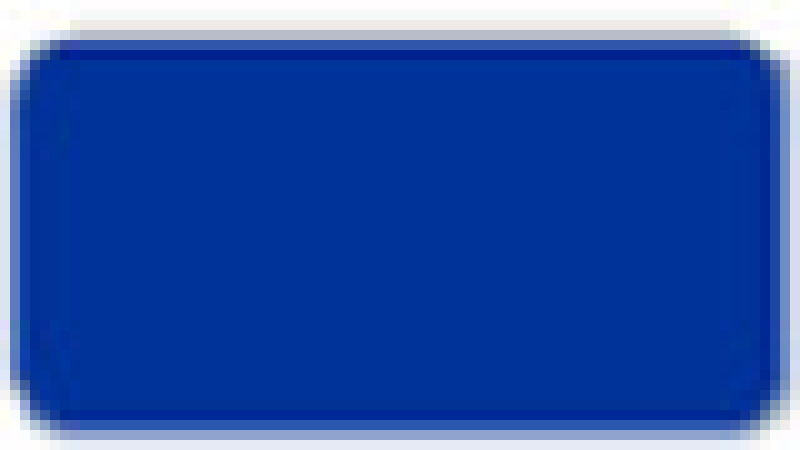
 FIST; 
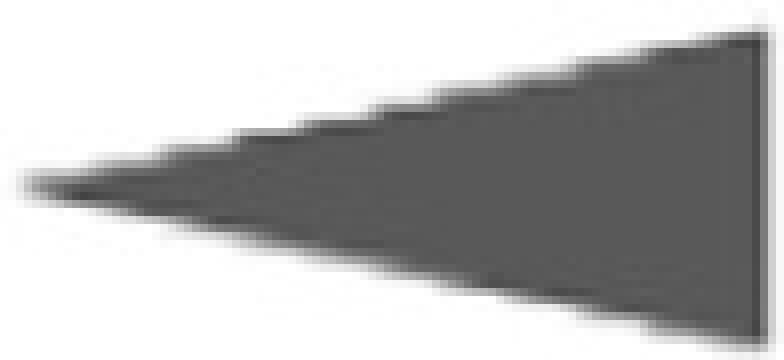
 HD-GYP. The individual structural domains represented by different colors and shapes are shown at the bottom. The shapes of all representative domains are not drawn to scale. For proteins containing GGDEF or EAL domain, the corresponding amino acid residues are written in black if they are not mutated, otherwise they are written in bold black. For predicted allosteric inhibition site (I_s_) of DGCs, √ means I_s_ included, otherwise not included.

We analyzed the neighboring genes of all 63 c-di-GMP metabolic proteins, since the possible transcriptional co-regulation at adjacent or proximate locations may affect their regulatory roles (Seshasayee et al., 2010; Schäper et al., 2017). On the basis of genome mapping and functional annotation, we identified 8 c-di-GMP metabolic proteins (VA3192, VA3193, VA4663, VA1419, VA3614, VA1297, VA3814 and VA2701) with adjacent or proximity operons encoding proteins that may be associated with biofilm formation or motility (Supplementary Figure S1). Notably, the neighboring genes of the GGDEF-only proteins VA4663 and VA1419 were predicted to be involved in biofilm formation, whereas the neighboring genes of the EAL-only protein VA3614 and the hybrid protein VA1297 (with an incomplete GGDEF) were predicted to be associated with motility. Furthermore, we found that some of the c-di-GMP metabolic proteins, including VA2972, VA2973, VA2974 and VA2975, VA1618 and VA1619, VA4622 and VA4624, as well as VA4030 and VA4033, are genomically encoded in close proximity to each other (Supplementary Figure S1). Previous studies have demonstrated that certain protein interactions between GGDEF/EAL domain proteins can specifically affect distinct output functions, and these proteins have to be co-expressed (Lindenberg et al., 2013; Sarenko et al., 2017). Based on these data, we speculate that these c-di-GMP metabolic proteins, which are closely encoded in the genome of *V. alginolyticus*, may benefit in co-regulating specific output functions through protein–protein interaction patterns.

The N-terminal sensory input domains located in the GGDEF or EAL has been shown to have a significant effect on the activity of DGCs or PDEs in response to environmental cues (Townsley and Yildiz, 2015). Thus, we analyzed the N-terminal sensory domains of all 63 c-di-GMP metabolic proteins. The PAS domain, which acts as a signal transducer coupling ligand binding to cellular signaling responses (Hutchin et al., 2021), was the most abundant N-terminal sensory domains and was observed in 12 c-di-GMP metabolic proteins (Table 1; Supplementary Table S3). Some even contain three PAS domains, such as the protein VA0461. Conserved domains with important roles in signal transduction, including HAMP, CHASE, REC and GAF, were also observed at the N-terminal of several c-di-GMP signaling proteins. Other N-terminal sensory domains detected in only one or two c-di-GMP metabolic proteins include PBPb, TPR, CBS, and FIST (Table 1; Supplementary Table S3).

The presence of transmembrane (TM) helices may be one of the reasons why distinct GGDEF (or EAL) proteins have different functions owing to their different intracellular localization (Guvener and Harwood, 2007; Collins et al., 2020; Hengge, 2021). The results showed that a total of 32 proteins contain TM domain(s) (Supplementary Figure S2; Supplementary Table S3), suggesting that these 32 proteins may be directly involved in the sensing of extracellular signals.

the highly conserved amino acid residues based on RxGGDE/EF motif are essential for DGC activity, while ExLxR or HD-GYP are required for PDE activity (Romling et al., 2013). Based on this proven theory, 29 of the 32 GGDEF-only proteins were predicted to have DGC activity (Supplementary Figure S3), while 10 of the 11 EAL-only proteins (Supplementary Figure S4) and 3 of the 4 HD-GYP-only proteins (Supplementary Figure S5) were predicted to possess PDE activity. In addition, 8 and 13 of the 16 hybrid proteins were predicted to have DGC and PDE activities, respectively, with 7 of them (VA0068, VA0088, VA0108, VA3608, VA3251, VA4309 and VA2417) having dual DGC and PDE activities. The remaining 2 hybrid proteins were predicted to be non-catalytic at the GGDEF and EAL sites, with VA3814 (LapD) and VA2701 (CsrD) predicted to be c-di-GMP receptors that may positively contribute to biofilm formation (Kitts et al., 2019; Kharadi and Sundin, 2022). For GGDEF domain-containing proteins with the RxxD motif, the presence of an allosteric product inhibition site (I-site) and its binding to c-di-GMP will result allosteric inhibition of DGC activity (Christen et al., 2006). Our results showed that nearly one-third of the GGDEF-only proteins contain the I-site, while only 1 of the 16 hybrid proteins contains the I-site (Table 1). Sequence alignment of all 63 c-di-GMP metabolic proteins with other GGDEF/EAL/HD-GYP domain-containing proteins revealed that the majority of residues in the active sites are conserved (Supplementary Figures S3–S6).

### Selection of 22 representative c-di-GMP metabolic proteins for functional characterization

**Figure 1:**
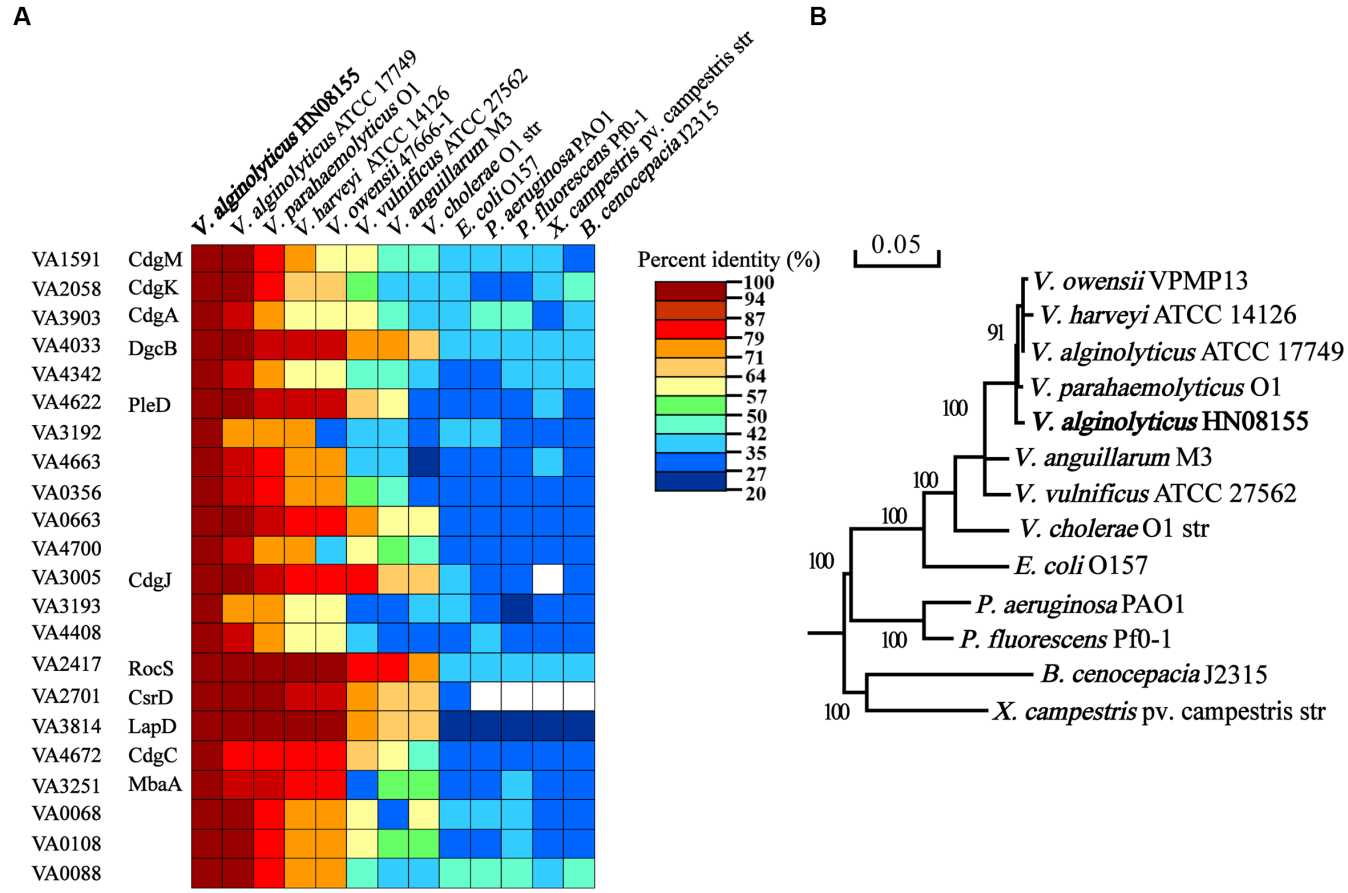
Comparisons of the 22 c-di-GMP metabolic proteins of *Vibrio alginolyticus* containing a GGDEF or EAL domain with those of closely related *Vibrio* species and other bacterial species **(A)**. Phylogenetic tree analysis of *V. alginolyticus* and other species based on 16S rRNA gene sequences **(B)**.

To investigate the regulatory role of c-di-GMP signaling in the control of biofilm-associated phenotypes in *V. alginolyticus*, 22 representative c-di-GMP metabolic genes (Figure 1) were selected for further study by the following three criteria. Firstly, given that some homologous genes have been reported to have regulatory roles in biofilm-associated phenotypes in other bacteria, 10 genes were selected based on this principle, including genes *VA1591* (*cdgM*), *VA2058* (*cdgK*), *VA3903* (*cdgA*), *VA4033* (*dgcB*), *VA3005* (*cdgJ*), *VA2417* (*rocS*), *VA2701* (*csrD*), *VA4672* (*cdgC*), *VA3251* (*mbaA*), and *VA4622* (*pleD*). Secondly, we selected 4 genes (*VA3192*, *VA3193*, *VA4663* and *VA3814*) whose neighboring genes encode proteins that may be associated with EPS production, biofilm formation, or motility (Supplementary Figure S1). In particular, the gene *VA3814* was also selected because it encodes a potential c-di-GMP receptor protein that shares 66.51% identity with the LapD protein of *V. cholerae* (Figure 1A), which has been demonstrated to regulate biofilm formation by controlling the adhesion protein LapA (Kitts et al., 2019). Finally, 3 genes encoding GGDEF-only proteins (*VA4342*, *VA0356* and *VA0663*), 2 genes encoding EAL-only proteins (*VA4700 VA4408*), and 3 genes encoding hybrid proteins (*VA0068*, *VA0108* and *VA0088*) were also selected based on the active or sensor domains they contain. The 22 c-di-GMP metabolic proteins of *V. alginolyticus* are conserved among closely related *Vibrio* species, including *V. parahaemolyticus*, *V. harveyi*, *V. owensii*, *V. vulnificus*, *V. cholerae*, and *V. anguillarum*, however, the conservativeness was significantly decreased in some phylogenetically distantly related bacterial species, such as *P. aeruginosa*, *P. fluorescens*, *Xanthomonas campestris* pv. *oryza*, *Burkholderia cenocepacia* and *E. coli* (Figure 1). Therefore, these 22 c-di-GMP metabolic proteins were selected as representatives and their individual functions in regulating biofilm-associated phenotypes were explored.

A total of 44 derivative strains with each of the 22 DGC or PDE genes knocked out or overexpressed were generated. As shown in Supplementary Figure S7, neither individual mutations nor overexpression of these 22 genes affected the growth of strain HN08155, so the phenotypic differences between the derivative and wild-type strains described below should be attributed to the regulatory role of the corresponding c-di-GMP metabolic genes.

### Effect of different c-di-GMP metabolic genes on colony morphology

**Figure 2:**
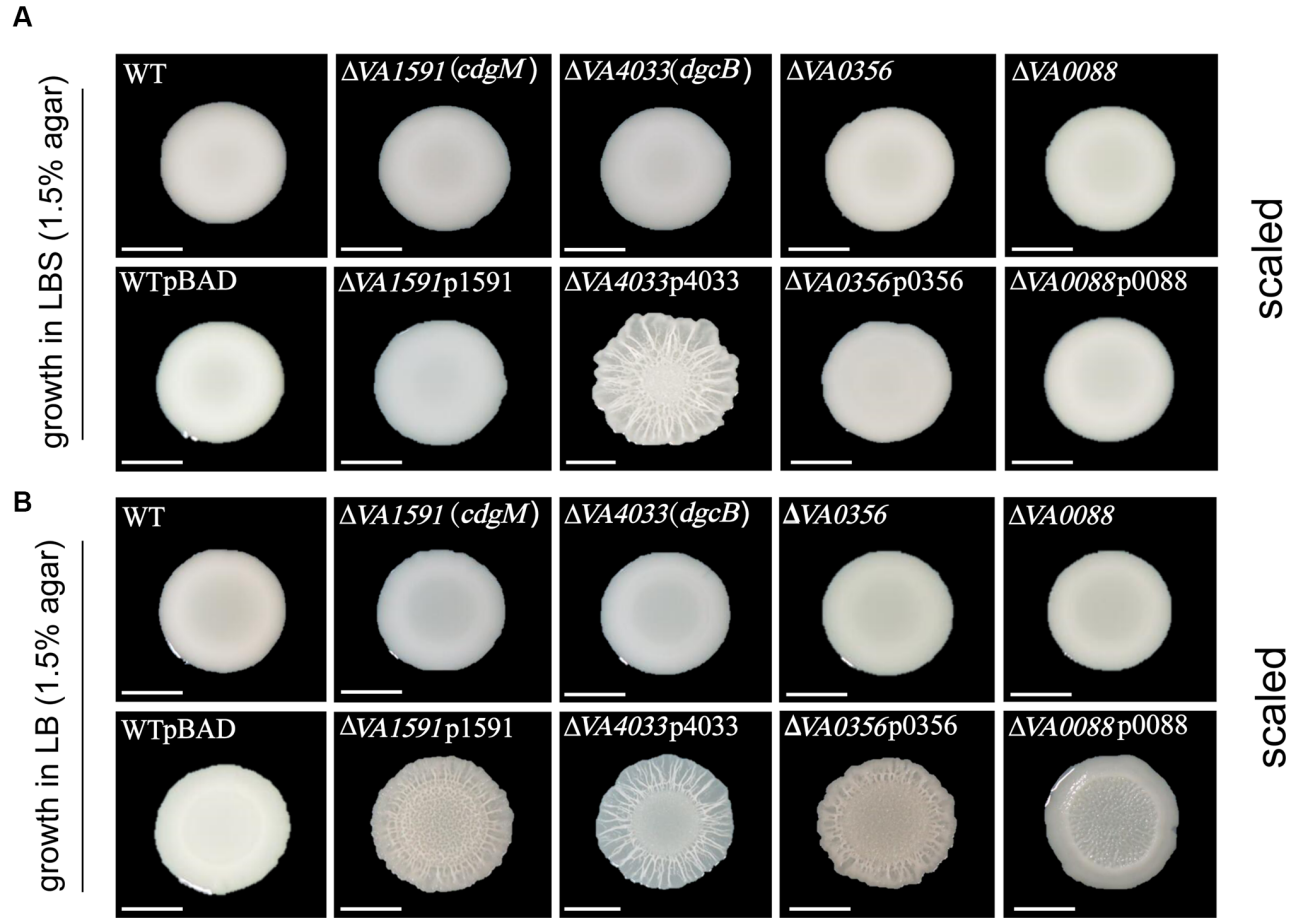
Representative images of colony morphology of the wild-type strain (or wild-type carrying pBAD vector) and 4 c-di-GMP metabolic gene deletion mutants and corresponding 4 overexpression strains on LBS **(A)** and LB **(B)** plates. Experiments were performed in three independent biological replicates and representative images are shown (scaled to equal diameter; bars = 5 mm).

Changes in colony morphology have been widely used to reflect changes in biofilm matrix production levels, as rugose (also termed wrinkled or corrugated) colony morphologies often correlate with increased EPS production (Yildiz and Visick, 2009). Thus, the regulatory effect of these 22 c-di-GMP metabolic genes on rugose colony formation was examined on LBS (containing 3% NaCl) and LB (containing 1% NaCl) plates (Supplementary Figure S8). We found that only one overexpression strain ∆*VA4033*p4033 exhibited rugose colony morphotypes on both LBS and LB plates (Figure 2A). Unlike strain ∆*VA4033*p4033, the overexpression strains ∆*VA1591*p1591, ∆*VA0356*p0356, and ∆*VA0088*p0088 only formed enhanced rugose colonies on LB plates (Figure 2B). The above results indicated that the regulatory effects of different c-di-GMP metabolic genes on the rugose colony morphology of *V. alginolyticus* are influenced by the salinity concentration in the medium. It is notable that all knockout mutant strains of the c-di-GMP metabolic gene had a smooth colony morphology similar to that of the wild-type (Supplementary Figure S8), indicating that the mutation of a c-di-GMP metabolic gene does not lead to changes in the colony morphology of *V. alginolyticus* under the assay conditions of this work.

### Correlation analysis of colony rugosity with EPS production and flagellar biosynthesis

**Figure 3:**
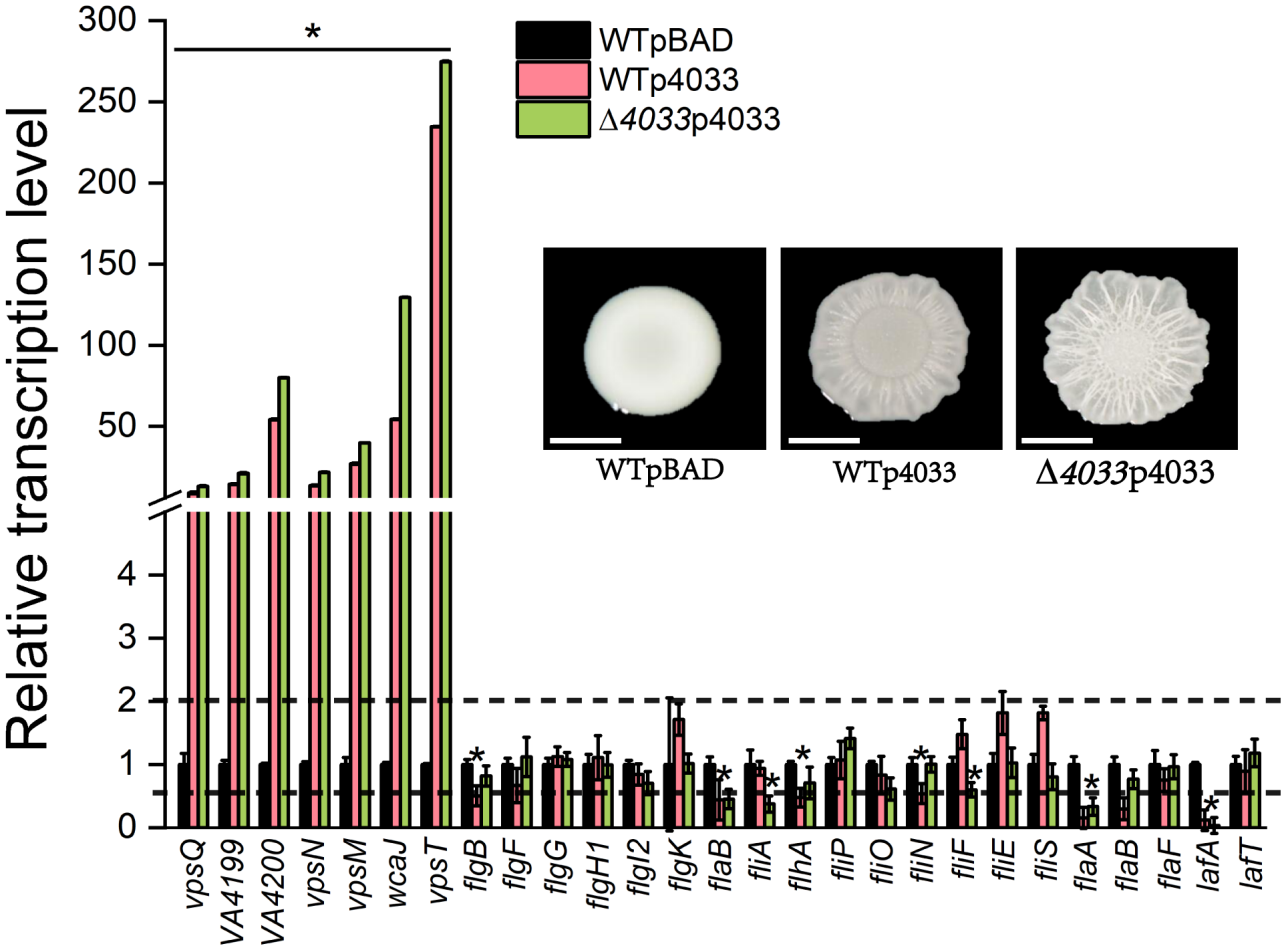
Relative expression levels of genes involved in EPS production and flagellar synthesis between strain WTpBAD and overexpression strains WTp4033 (or ∆*VA4033*p4033) on LBS plates. The transcript levels of genes in WTpBAD were set to a value of 1 as a reference, and the upper and lower dashed lines indicate expression levels of 2-fold and 50% compared to the WTpBAD, respectively. The results presented are the mean of triplicate experiments and error bars represent SDs. *, *p* < 0.05.

The DGC VA4033 (DgcB) was the only one of the 22 representative c-di-GMP metabolic proteins that enhanced colony rugosity on both LBS and LB plates. For this reason, correlations between colony rugosity with EPS production and flagellar analysis were determined by quantitative PCR (qPCR) under conditions of overexpression of VA4033. As shown in Figure 3, overexpression of VA4033 significantly increased the transcript levels of all tested genes that are involved in EPS production, including 6 genes of the EPS biosynthetic gene cluster (*vpsQ*-*wcaJ*), as well as the *vpsT* gene encoding a helix-turn-helix transcriptional regulator (*p* < 0.05). On the contrary, most of the genes in the flagellar synthesis gene cluster, including class I (*flgBFGH1I2K*-*flaB*), class II (*fliA*-*flhA*-*fliPON*), class III (*fliFES*-*flaABF*) and *lafAT*, were significantly down-regulated in the overexpression strains WTp4033 and ∆*4033*p4033 compared to strain WTpBAD (*p* < 0.05; Figure 3). The above results indicate that rugosity morphology is accompanied by increased transcript levels of EPS biosynthesis genes and decreased expression levels of flagellar synthesis genes, suggesting that c-di-GMP inversely regulates EPS production and flagellar biogenesis in *V. alginolyticus*.

### Identification of c-di-GMP metabolic genes involved in static biofilm formation

To determine which c-di-GMP metabolic genes play a role in biofilm formation in *V. alginolyticus*, we quantified the static biofilm formation ability of c-di-GMP metabolic gene deficient and overexpression strains under LB culture condition by crystal violet staining. Our previous results showed that the biofilm formation ability of the mutant strain ∆*cdgH* was significantly lower than that of the wild-type, and therefore the biofilm-deficient strain ∆*cdgH* was used as a negative control in all subsequent biofilm assays.

**Figure 4:**
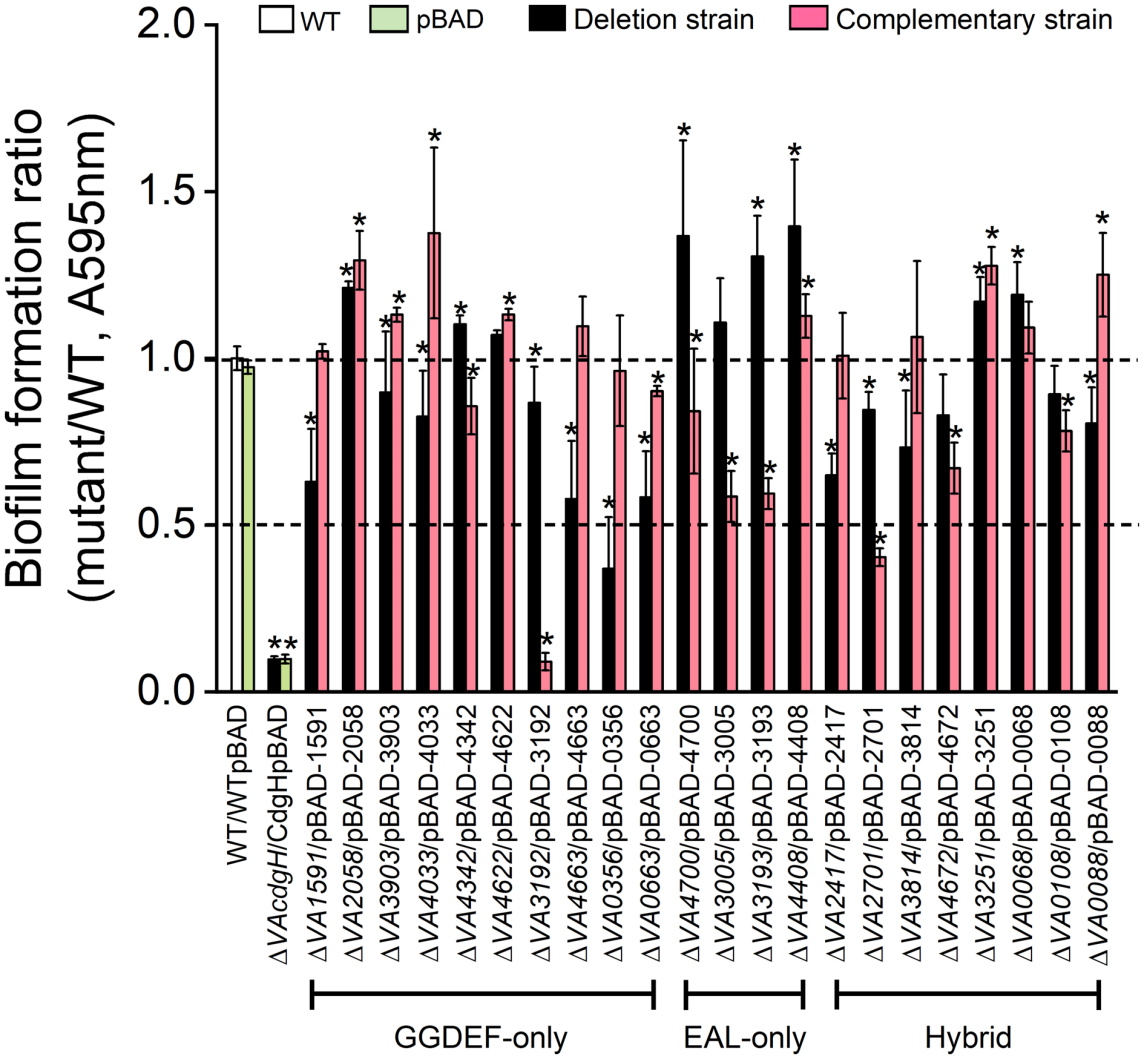
Static biofilm formation assay. The wild-type, 22 c-di-GMP metabolic gene deletion mutants, and the corresponding 22 overexpression strains were cultured in LB liquid medium in 96-well plate for 6 h. All measurements of biofilm biomass were normalized to the value of the wild-type. The results presented are the mean of triplicate experiments and error bars represent SDs. *, *p* < 0.05.

Among the 10 mutants of GGDEF-only gene, 6 of them (∆*VA1591*, ∆*VA3903*, ∆*VA4033*, ∆*VA4663*, ∆*VA3192* and ∆*VA0663*) formed biofilms at approximately 60% ~ 90% of the level of the wild-type, 1 mutant (∆*VA0356*) formed biofilms at about 40% of the level of the wild-type, and 3 mutants (∆*VA2058*, ∆*VA4342* and ∆*VA4622*) formed biofilms at levels exceeding that of the wild-type (Figure 4). The biofilm formation levels of the corresponding overexpression strains of the 10 c-di-GMP metabolic genes recovered to varying degrees to that of the wild-type, with strains ∆*VA1591*p1591 and ∆*VA0356*p0356 being almost comparable to the wild-type, strains ∆*VA0663*p0663 and ∆*VA4342*p4342 producing slightly lower biofilms than the wild-type, and strains ∆*VA2058*p2058, ∆*VA3903*p3903, ∆*VA4033*p4033, ∆*VA4622*p4622 and ∆*VA4663*p4663 formed biofilms slightly or even significantly higher than the wild-type (Figure 4). Unexpectedly, despite the fact that VA3192 was predicted to be a DGC and its GGDEF domain was intact, the biofilm formation level of strain ∆*VA3192*p3192 was as low as that of the negative control *∆cdgH*. We speculate that a possible explanation is that the protein VA3192 may not be involved in the regulation of biofilm formation by c-di-GMP metabolism, as previous study has indicated that the regulation of biofilm formation by the protein GdpS in *Staphylococcus* was independently of c-di-GMP (Holland et al., 2008). In conclusion, these results indicate that most of the predicted DGCs can promote static biofilm formation, and their mode of action is likely to be through the synthesis of c-di-GMP.

All of the 4 mutants of EAL-only gene, including *∆VA4700*, ∆*VA3005*, ∆*VA3193* and ∆*VA4408*, showed significantly increased biofilm formation compared to the wild-type, with mutant ∆*VA3005* producing a lower biofilm than the other three mutants. The corresponding overexpression strains ∆*VA4700*p4700, ∆*VA3005*p3005 and ∆*VA3193*p3193 formed biofilms at approximately 50% of the level of the wild-type, however, strain ∆*VAVA4408*p4408 showed no deficiency in biofilm formation (Figure 4).

Six of the 8 mutants of GGDEF-EAL gene (∆*VA2417*, ∆*VA2701*, ∆*VA3814*, ∆*VA4672*, ∆*VA0108* and ∆*VA0088*) formed biofilms at 50% ~ 90% of the level of the wild-type. The level of biofilms formed by the overexpression strains ∆*VA2417*p2417 and ∆*VA3814*p3814 was close to the wild-type, and the biofilm formation ability of strain ∆*VA0088*p0088 was significantly higher than that of the wild-type (*p* < 0.05), probably because the DGC activity of protein VA0088 of *V. alginolyticus* is prevalent under the assay conditions of this work. The level of biofilms formed by the overexpression strains ∆*VA2701*p2701, ∆*VA4672*p4672 and ∆*VA0108*p0108 was significantly lower than that of the wild-type (*p* < 0.05). The remaining two mutants ∆*VA3251* and ∆*VA0068* exhibited significantly higher biofilm formation ability than the wild-type (*p* < 0.05), and their corresponding overexpression strains ∆*VA3251*p3251 and ∆*VA0068*p0068 also showed enhanced biofilm formation ability compared to the wild-type (Figure 4).

### Identification of c-di-GMP metabolic genes involved in EPS production

The intracellular c-di-GMP level is positively correlated with EPS production (Rashid et al., 2003; Casper-Lindley and Yildiz, 2004; Srivastava et al., 2013), and the ability of EPS to bind Congo Red (CR) has been shown to be an effective and intuitive way to detect EPS (Schäper et al., 2017). Therefore, we identified the c-di-GMP metabolic genes involved in EPS production in *V. alginolyticus* by CR binding method.

**Figure 5:**
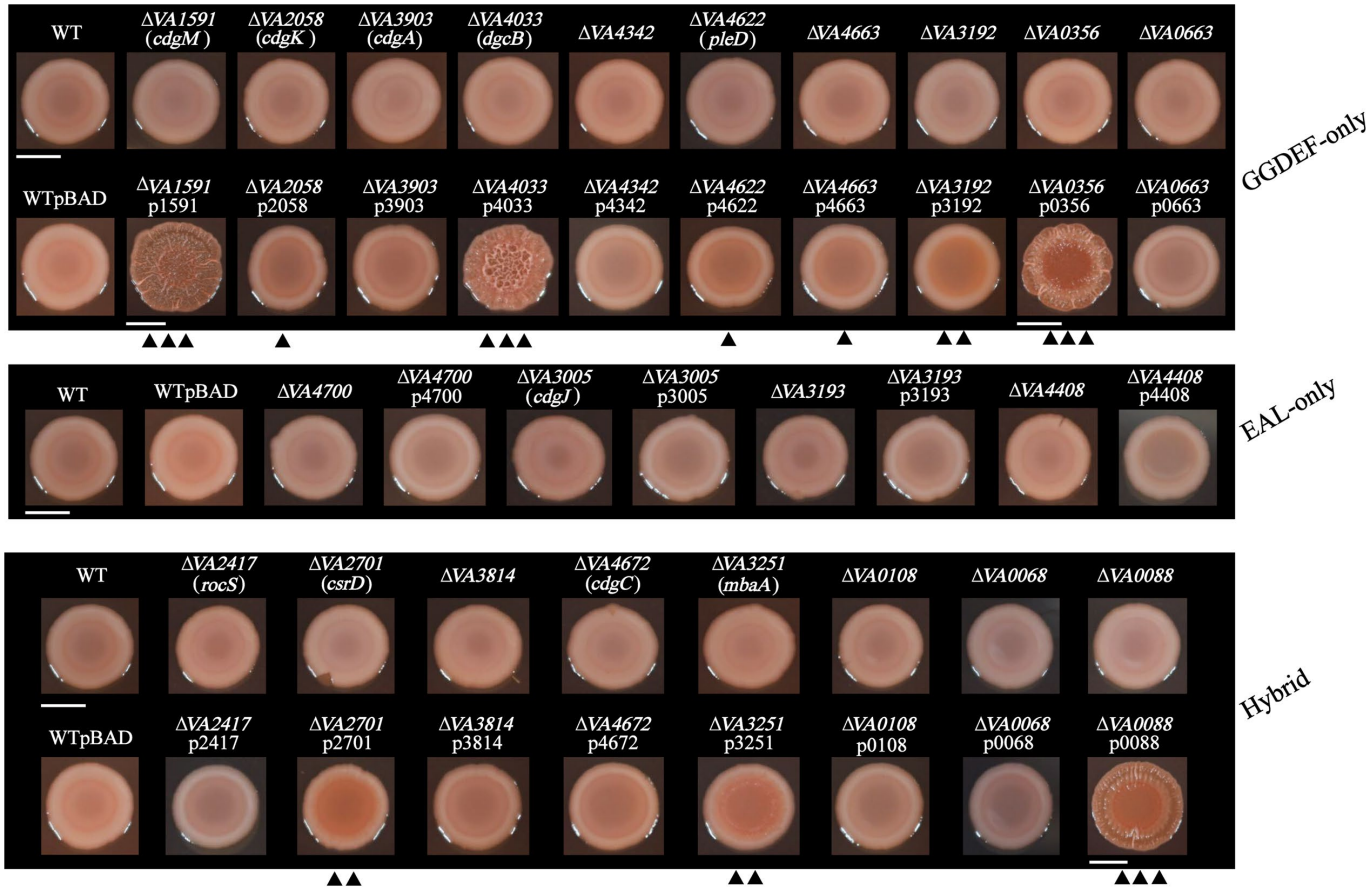
EPS production determined with Congo red plates. Representative images of the wild-type, 22 c-di-GMP metabolic gene deletion mutants and corresponding 22 overexpression strains on TSA plates staining with Congo red were shown. Experiments were performed in three independent biological replicates (scaled to equal diameter; bars = 5 mm).

Among the 10 mutants of GGDEF-only gene, none of them showed a significant difference in CR binding intensity from that of the wild-type (Figure 5), suggesting that deletion of a single c-di-GMP metabolic gene cannot significantly cause changes in CR binding activity. However, overexpression strains of these 10 DGCs resulted in significant changes in CR binding ability, with strains ∆*VA1591*p1591, ∆*VA4033*p4033, and ∆*VA0356*p0356 showing strong CR binding ability and rugose colony morphologies, and strain ∆*VA3192*p3192 showing lower CR binding than the above three strains, followed by ∆*VA2058*p2058, ∆*VA4622*p4622, and ∆*VA4663*p4663, and the remaining 3 overexpression strains ∆*VA3903*p3903, ∆*VA4342*p4342, ∆*VA0663*p0663 had similar CR binding ability to the wild-type (Figure 5).

Similarly, none of the 4 mutants of EAL-only gene (∆*VA4700*, ∆*VA3005*, ∆*VA3193*, and ∆*VA4408*) displayed a significant difference in CR binding ability from that of the wild-type (Figure 5). However, unlike most of the DGC overexpression strains showed stronger CR binding than the wild-type, none of the 4 PDE overexpression strains (∆*VA4700*p4700, ∆*VA3005*p3005, ∆*VA3193*p3193, and ∆*VA4408*p4408) had weaker CR binding compared to the wild-type (Figure 5).

All of the 8 mutants of GGDEF-EAL gene (∆*VA2417*, ∆*VA2701*, ∆*VA3814*, ∆*VA4672*, ∆*VA3251*, ∆*VA0068*, ∆*VA0108*, and ∆*VA0088*) had indistinguishable CR binding compared to the wild-type (Figure 5). Compared to the wild-type, the overexpression strain ∆*VA0088*p0088 exhibited stronger CR binding and enhanced colony rugosity (Figure 5), which was consistent with its increased static biofilm formation ability (Figure 4). The overexpression strains ∆*VA2701*p2701 and ∆*VA3251*p3251 also showed higher CR binding ability than the wild-type, but displayed smooth colony morphology (Figure 5). The remaining overexpression strains ∆*VA2417*p2417, ∆VA*4672*p4672, ∆*VA0068*p0068, and ∆*VA0108*p0108 showed similar CR binding ability to the wild-type (Figure 5).

### C-di-GMP metabolic proteins were essential for flagellar-mediated swarming motility

**Figure 6:**
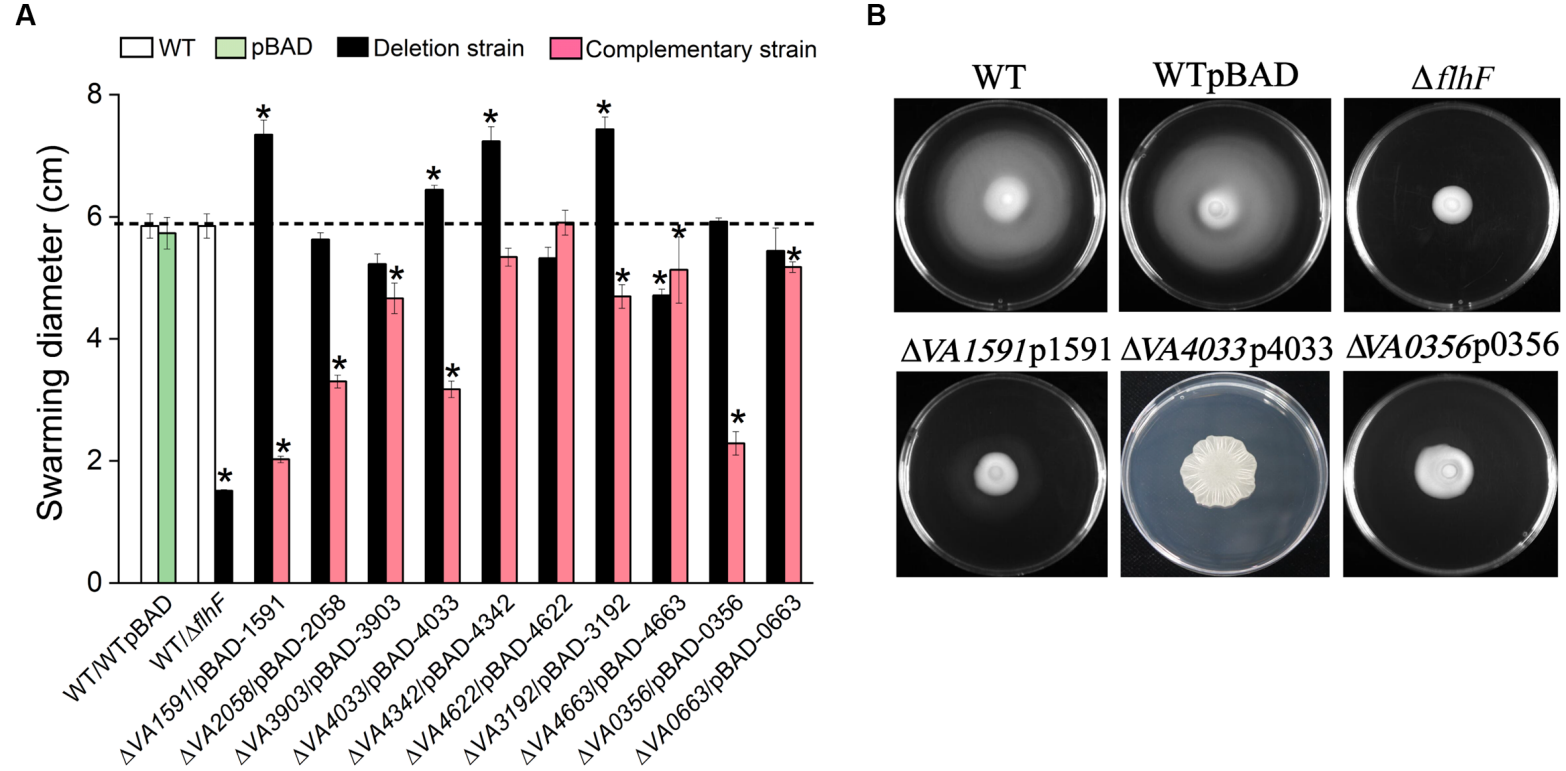
Swarming motility assay. The swarming diameter of the wild type, 10 c-di-GMP metabolic gene deletion mutants, and corresponding 10 overexpression strains on LBS plates with 0.6% agar, the results presented are the mean of triplicate experiments and error bars represent SDs. *, *p* < 0.05. **(A)** Representative images of swarming motility reduction in strains ∆*1591*p1591, ∆*4033*p4033 and ∆*0356*p0356. The nonmotile (no clearly visible movement outside the colony) ∆*flhF* mutant served as a negative control **(B)**.

The swarming motility and biofilm formation or EPS production are inversely regulated by c-di-GMP-mediated signaling (Kuchma et al., 2007, 2015). Semi-solid motility plates containing 0.6% agar were used to investigate whether the 10 proteins involved in the synthesis of c-di-GMP play a role in flagellar-mediated swarming motility. The ∆*flhF* strain cannot synthesis flagella as a negative control. Of the 10 mutants of GGDEF-only gene, 4 of them (∆*VA1591*, ∆*VA4033*, ∆*VA4342* and ∆*VA3192*) showed a significant increase in swarming diameter compared to the wild type. In contrast, mutants ∆*VA2058*, ∆*VA3903*, ∆*VA4622*, ∆*VA0356*, ∆*VA0663*, and ∆*VA4663* showed a slight decrease in swarming diameter compared to the wild type or were comparable to the wild type (Figure 6A). The respective overexpression strains of these mutants produced varying degrees swarming inhibition, with strains ∆*VA1591*p1591 and ∆*VA0356*p0356 producing less than 50% of the swarming phenotype compared to the wild type, strains ∆*VA2058*p2058 and ∆*VA4033*p4033 showing 50% ~ 60% of the swarming ability level of the wild type, and strain ∆VA4033p4033 producing a rugose phenotype (Figure 6B). Strains ∆*VA3903*p3903, ∆*VA3192*p3192, ∆*VA4342*p4342, ∆*VA4663*p4663, and ∆*VA0663*p0663 exhibited swarming ability at 70% ~ 90% level of the wild type, while strain ∆*VA4622*p4622 had comparable swarming ability to the wild type (Figure 6A). These results suggest that these 10 predicted DGCs have different degrees of inhibition on the swarming ability of *V. alginolyticus*.

## Discussion

**Figure 7:**
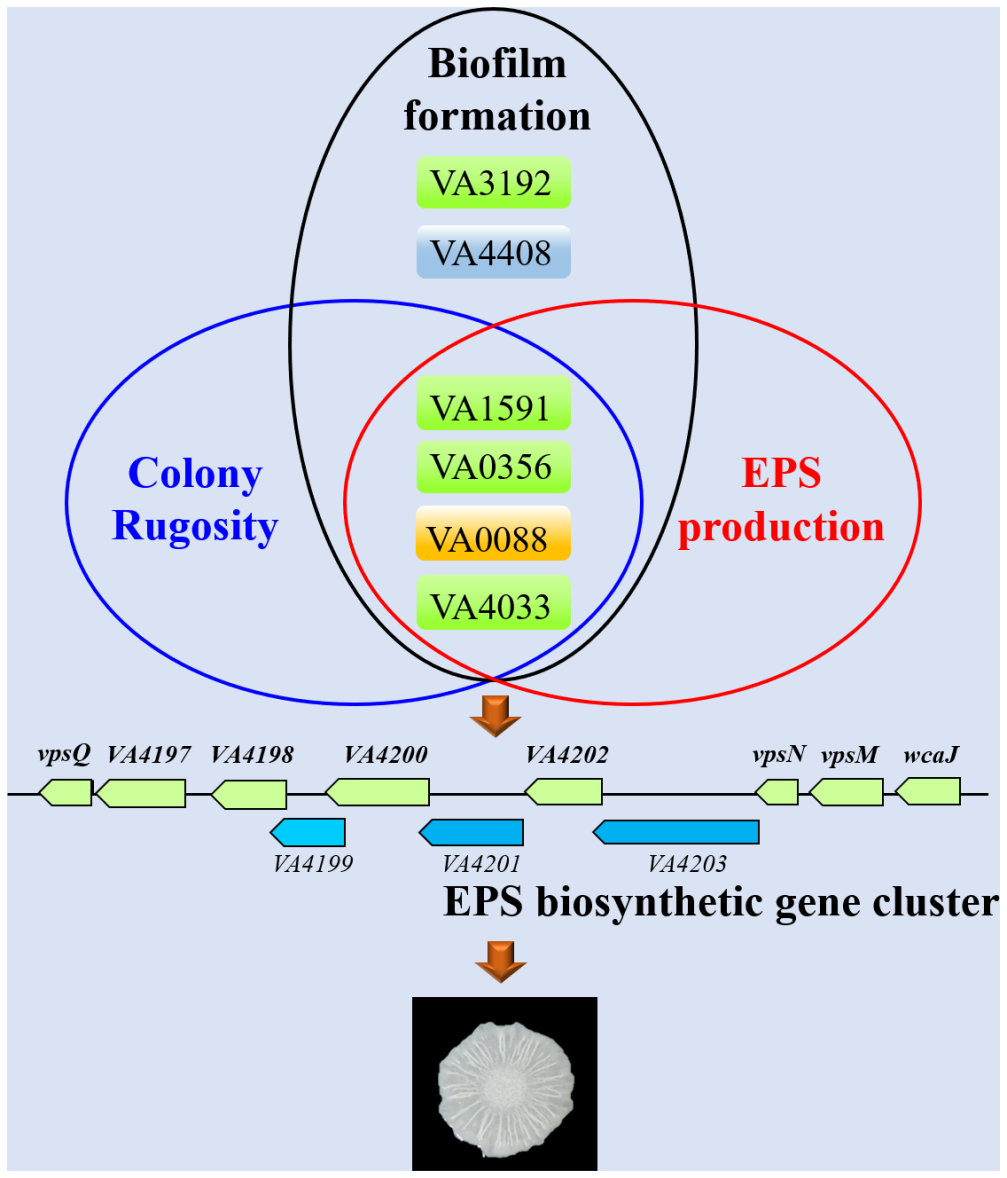
Schematic for proposed conceptual model for key c-di-GMP metabolic genes that required for biofilm-associated phenotypes in *V. alginolyticus*.

Currently, it is still unclear about the role of c-di-GMP signaling in *V. alginolyticus* and how the different c-di-GMP metabolic proteins regulate biofilm formation. In this work, we identified a total of 63 c-di-GMP metabolic proteins with putative GGDEF/EAL/HD-GYP domains in the genome of *V. alginolyticus* HN08155. By systematic bioinformatic analyses, we selected 22 representative c-di-GMP metabolic proteins and explored their regulatory roles in biofilm-associated phenotypes, including colony rugosity, static biofilm formation, EPS production and motility. To this end, we constructed knockout mutants and overexpression strains of these 22 c-di-GMP metabolic genes, respectively, and found that several key c-di-GMP metabolic genes (*VA1591*, *VA0356*, *VA0088*, *VA4033*, *VA3192* and *VA4408*) had significant influences on the biofilm-associated phenotypes of *V. alginolyticus* (Figure 7).

We identified 4 c-di-GMP metabolic genes, including *VA4033*, *VA1591*, *VA0356*, and *VA0088*, all with intact active sites for the GGDEF domain (Table 1), involved in the enhanced rugose colony phenotypes on LB agar plates (Figure 2). It is well known that there is a strong correlation between the phenotypes of colony rugosity, biofilm formation and EPS production, and moreover, these phenotypes are often accompanied by elevated intracellular c-di-GMP levels (Nakhamchik et al., 2008; Romling et al., 2013; Wu et al., 2020; Xiao et al., 2022). In general, enhanced EPS production often results in rugose colony phenotypes (Casper-Lindley and Yildiz, 2004; Beyhan et al., 2008), a phenomenon also demonstrated in the present work, for example, overexpression strains ∆*VA1591*p1591, ∆*VA4033*p4033, ∆*VA0356*p0356, and ∆*VA0088*p0088 produced rugose colonies that also had strong CR binding capacities (Figures 2, 5). However, in some strains, these phenotypes were only weakly correlated. For example, overexpression strains ∆*VA1591*p1591 and ∆*VA0356*p0356 had a corrugated phenotype and strong CR binding but did not produce a hyper-biofilm phenotype, in contrast, some strains (e.g., ∆*VA2058*p2058, ∆*VA4700*, ∆*VA3005*, ∆*VA3193*, and ∆*VA4408*) had significantly increased biofilm formation but produced smooth colony morphology with weak binding to CR (Supplementary Figure S8; Figures 4, 5). Furthermore, overexpression strains ∆*VA3192*p3192 and ∆*VA2701*p2701 showed relatively stronger binding ability to CR than the wild-type, but produced a significant deficiency in biofilm formation (Figures 4, 5). The deficiency of biofilm formation in strain ∆*VA3192*p3192 was inconsistent with the predicted DGC activity of the protein VA3192, although this phenomenon has been previously identified (Sperling et al., 2019), however, whether this inconsistency is mediated by c-di-GMP signaling will be further investigated. In summary, colony rugosity and CR binding can be largely indicative of each other, especially strong CR binding that concomitantly characterizes the phenotype of super rugose. However, biofilm formation does not always correspond to the above two phenotypes. As previously reported, the functional diversity of c-di-GMP metabolic proteins in other Gram-negative bacteria has also been demonstrated (Ha et al., 2014; Chen et al., 2020; Shrestha et al., 2022).

Several previous studies have demonstrated that changes in the expression patterns of genes associated with EPS biosynthetic cluster are of significance for the biofilm structure of *Vibrio* species (Fong and Yildiz, 2007; Fong et al., 2017). We identified a cluster of 11 genes that encode proteins predicted to be associated with the production of EPS in *V. alginolyticus* HN08155, and several proteins in this cluster are homologous to the *vps* cluster of *V. cholerae* (Figures 3, 7). VpsT is a master transcriptional regulator that activates the expression of genes in the *vps* cluster by binding to the promoter sequence of *vpsL* in a c-di-GMP-dependent manner (Casper-Lindley and Yildiz, 2004; Tischler and Camilli, 2004; Krasteva et al., 2010). In this study, we demonstrated that the transcript levels of several genes in the EPS biosynthetic cluster were significantly increased in rugose colonies (WTp4033 and ∆*VA4033*p4033) compared to smooth colonies (WTpBAD), which is consistent with previous findings that EPS production leads to rugose morphology (Yildiz et al., 2001; Rashid et al., 2003; Casper-Lindley and Yildiz, 2004; Beyhan et al., 2008; Teschler et al., 2015).

This study showed that most of the mutants of the GGDEF-only proteins had increased or equivalent swarming motility phenotypes to the wild type, in contrast, 9 out of 10 DGC overexpression strains were inhibited in swarming motility (Figure 6). This was in accordance with the established research findings that high c-di-GMP levels suppress motility (Liu et al., 2010; Romling et al., 2013; Conner et al., 2017). Interestingly, the overexpression strain ∆*VA4033*p4033 exhibited a unique phenotype of weak and flat corrugated colonies on plate containing 0.6% agar, which was totally different from the smooth extended colonies of the other 9 overexpression strains on the same soft agar plate (Figure 6B; Supplementary Figure S9). In *Caulobacter crescentus*, the DGC activity of DgcB is stimulated upon surface sensing, a process mediated by flagellar motor, which ultimately leads to the rapid synthesis of polysaccharide adhesins called the holdfast (Hug et al., 2017). In this study, the qPCR results showed that the transcript levels of EPS biosynthesis genes were significantly increased when the function of protein VA4033 (DgcB) was fully complemented (data not shown). In addition, Hershey et al. found that DgcB could produce c-di-GMP at an instantaneous burst rate and immediately stimulate the production of polysaccharide adhesins in *C. crescentus* (Hershey et al., 2021). Therefore, although the plate containing 0.6% agar is favorable for flagellar-mediated motility (Supplementary Figure S9), overexpression strain ∆*VA4033*p4033 can also produce flat corrugated colonies, which we speculate is due to its “fast nature” in producing c-di-GMP and polysaccharide adhesins. However, the specific regulatory mechanism of protein VA4033 on the flat colony rugosity of *V. alginolyticus* under semi-solid condition needs further investigation.

In summary, this work provides the first insight into the role of c-di-GMP metabolic genes in controlling colony morphology, biofilm formation, EPS production and motility in *V. alginolyticus*. We found that each c-di-GMP metabolic gene can regulate multiple phenotypes, with 4 key c-di-GMP metabolic genes having important roles in the control of colony rugosity and EPS production. The large number of c-di-GMP metabolic proteins encoded by *V. alginolyticus* suggests that this marine pathogen may utilize a complex c-di-GMP signaling regulatory network to modulate its lifestyle changes in response to various environmental cues.

## Materials and methods

### Bioinformatic analyses

The functional annotation results of *V. alginolyticus* HN08155 were obtained from 11 databases, including VFDB, ARDB, CAZY, IPR, SWISSPROT, COG, CARD, GO, KEGG, NR, and T3SS, from the genome sequencing dataset. The c-di-GMP metabolic proteins were retrieved from the result annotation file based on “search for conserved domains.” For all searches: the entry “GGDEF” refers to GGDEF domain, “EAL” refers to EAL domain, and “HD” refers to HD-GYP domain. The transmembrane signaling and sensory partner domains of all c-di-GMP metabolic proteins were analyzed using the SMART algorithm^1^ and NCBI’s Conserved Domain Database (CDD).^2^ Conservation analysis of the GGDEF and EAL domain proteins of *V. alginolyticus* with other bacterial species using the NCBI Blastp Database. The phylogenetic tree was constructed with DNAMAN software.

Sequence alignments of the GGDEF, EAL and HD-GYP domains of *V. alginolyticus* and other related species were generated with ClustalW^3^ and formatted with ESPript 3.0^4^ to determine the positions of the corresponding conserved site residues. Sequence logos were created based on the conserved domains of GGDEF, EAL and HD-GYP using the DNAMAN comparison tool and WebLogo 3: Create.^5^ The intactness of the catalytic sites of GGDEF, EAL and HD-GYP was determined according to previous research (Galperin et al., 1999; Christen et al., 2006; Seshasayee et al., 2010; Romling et al., 2013; Shrestha et al., 2022). Briefly, the active site (A_GGDEF_ site) in the GGDEF domain was considered functional if the RxGGD/EEF signature motif was unmutated, and the inhibitory site (I_GGDEF_ site) that can bind c-di-GMP was considered functional if an RxxD motif was present in 5 residues upstream of the A_GGDEF_ site. The A_EAL_ site was considered functional if the ExLxR signature motif was unmutated. The A_HD-GYP_ site was considered functional if the HHExxDGxxGYP motif was conserved.

### Strains and growth conditions

Bacterial strains and plasmids used in this study are listed Supplementary Table S1. *V. alginolyticus* strains were grown in Luria-Bertani (LB) medium (1% tryptone, 0.5% yeast extract, and 1% NaCl) or LBS medium (1% tryptone, 0.5% yeast extract, and 1% NaCl) at 30°C. *E*. *coli* used for DNA manipulation (strains DH5α and Top10) and for conjugational transfer (strain β2163) were routinely cultured in LB medium at 37°C. Solid medium was prepared using 1.5% agar. When needed, antibiotics were added at the following concentrations: for *E. coli*, ampicillin at 100 μg/mL and chloramphenicol at 50 μg/mL; and for *V. alginolyticus*, ampicillin at 100 μg/mL and chloramphenicol at 25/50 μg/mL for liquid/solid medium, respectively. L-arabinose at 0.02% (vt/vol) in growth medium was used for overexpression strains, and the final concentration of 2,6-Diaminopimelic acid (DAP) used for strain β2163 was 0.3 mM. Unless stated otherwise, all *V. alginolyticus* strains were pre-cultured in LBS medium containing appropriate antibiotics.

### Construction of mutants and overexpression strains

All DNA manipulations were performed by standard molecular protocols. Deletion mutants of interest genes in *V. alginolyticus* were constructed via allelic exchange system. Briefly, the up-and down-stream flanking regions of the desired deletion gene, each approximately 600 bp in length, were amplified separately from the genomic DNA of *V. alginolyticus* using the primers shown in Supplementary Table S2. The two PCR products were ligated by overlapping extension PCR technique and cloned into the suicide plasmid pDM4 via designed restriction enzyme sites using T4 DNA ligase. The recombinant plasmid was transformed into a conjugative strain β2163 containing the *pir* gene, which was then conjugated with *V. alginolyticus*. Transconjugants were selected on LBS agar plates containing both ampicillin and chloramphenicol. Finally, *V. alginolyticus* mutant strains that had undergone homologous recombination were selected with 10% sucrose and validated by PCR.

For the construction of overexpression strains, the coding region of interest genes (excluding the stop codon) was amplified and ligated into the plasmid pBAD/Myc-HisA (the original ampicillin resistance site was replaced by chloramphenicol) by One Step Cloning Kit (Vazyme, Nanjing, China). The recombinant plasmid was transformed into *E. coli* Top10, and after sequencing, the plasmid was transformed into the conjugative strain β2163. All of these constructs were expressed to produce myc-6xHis tagged recombinant proteins. All PCR products were validated by DNA sequencing.

### Analysis of bacterial growth

Freshly pre-cultured bacterial cultures were adjusted to the same cell density and inoculated 1:100 into fresh LBS or LB medium without antibiotics (supplemented with L-arabinose when needed), and then transferred 800 μL into each well of a 48-well microtiter plate. Fresh LBS or LB medium without bacterial inoculation served as a negative control. Bacterial growth was monitored by measuring OD_600_ every 30 min for 24 h at 30°C, using a multilabel plate reader (Biotek Winooski, Vermont, United States).

### Analysis of colony morphology

Colony morphology assays were performed on LBS and LB plates. A single colony of freshly cultured *V. alginolyticus* strains was inoculated and grown in liquid LBS medium with shaking for 12 h (supplemented with antibiotics when appropriate). Bacterial cultures in LBS medium were diluted 1:100 into 20 mL of fresh LBS medium (supplemented with antibiotics and 0.02% L-arabinose when appropriate), and shaken for 3–4 h, then cell density was measured and normalized to an OD_600_ of 0.7. Afterwards, 4 μL of the resulting culture was spotted on LBS or LB agar plates (containing 1.5% agar) without antibiotics, allowed to dry and incubated at 30°C for 24 h. The diameter of the colonies was measured using a vernier caliper and imaged using a Canon PowerShot G7 X Mark III camera.

### RNA extraction and qPCR

Colony biofilms of strains WTpBAD, WTp4033, and ∆*VA4033*p4033 were collected separately at 24 h and total RNA was extracted using Trizol reagent according to standard procedures. First-strand cDNA was synthesized using the HiScript® II Q RT SuperMix for qPCR (+gDNA wiper; Vazyme). Quantitative PCR (qPCR) was performed using the ChamQ Universal SYBR qPCR Master Mix (Vazyme) on a real-time PCR instrument (Roche, Basel, Switzerland). The relative expression levels of the target genes were calculated using the 2^-ΔΔCt^ method (Livak and Schmittgen, 2001). The *gyrB* gene was used as the internal reference gene. The data were analyzed based on three independent biological replicates.

### Biofilm formation assay

Biofilm formation assays were performed in LB medium according to previous research with minor modifications (Wilksch et al., 2011). Freshly pre-cultured bacterial cultures (adjusted to an OD_600_ of 0.75) were inoculated 1:100 into fresh LB medium without antibiotics but supplemented with L-arabinose, and then transferred 200 μL into each well of a 96-well microtiter plate. Fresh LB medium without bacterial inoculation served as a negative control. The plates were incubated at 30°C for 6 h under static conditions, at which point the cultures were discarded from the wells and washed twice with PBS buffer. Wells were dried and then stained with 0.1% crystal violet for 20 min, followed by slow washing with distilled water and subsequently allowed to dry. Wells were destained with 200 μL of 33% (vol/vol) acetic acid for 20 min, and then the solution was transferred to a new 96-well plate, followed by quantification by measuring the optical density at 595 nm on a multilabel plate reader (BioTek).

### Congo red assay

Congo red staining was performed on TSA agar plates containing 120 μg/mL Congo red (supplemented with 0.02% L-arabinose when appropriate). Five microliters of freshly pre-cultured bacterial cultures (adjusted to an OD_600_ of 0.7) was dropped onto TSA agar plates without antibiotics. The plates were incubated at 30°C for 24 h and left to stand at room temperature for about 12 h, and then imaged using a Canon PowerShot G7 X Mark III camera.

### Swarming motility assay

All *V. alginolyticus* strains were tested for swarming motility on LBS plates supplemented with 0.02% L-arabinose when appropriate. Two microliters of freshly pre-cultured bacterial cultures (adjusted to an OD_600_ of 0.7) was spotted on swarming plates containing 0.6% agar and incubated at 30°C for 24 h. The mutant strain ∆*flhF* without swarming ability was used as a negative control. The swarming motility assay was performed in at least six replicates of three independent experiments.

### Statistical analyses

Statistical analyses were carried out using the SPSS 18.0 statistical software. All assays were performed in triplicate, and the results were presented as the mean ± standard deviation. Analyses of statistical differences were conducted with the paired two-tailed Student’s *t* test.

### Data availability

The GenBank accession numbers of the 22 c-di-GMP metabolic genes, including *VA1591* (*cdgM*), *VA2058* (*cdgK*), *VA3903* (*cdgA*), *VA4033* (*dgcB*), *VA4342*, *VA4622* (*pleD*), *VA3192*, *VA4663*, *VA0356*, *VA0663*, *VA4700*, *VA3005* (*cdgJ*), *VA3193*, *VA4408*, *VA2417* (*rocS*), *VA2701* (*csrD*), *VA3814* (*lapD*), *VA4672* (*cdgC*), *VA3251* (*mbaA*), *VA0068*, *VA0108* and *VA0088* were OP893990, OP893991, OP893992, OP893993, OP893994, OP893995, OP893996, OP893997, OP893998, OP893999, OP894000, OP894001, OP894002, OP894003, OP894004, OP894005, OP894006, OP894007, OP894008, OP894009, OP894010, and OP894011, respectively.

## Data availability statement

The datasets presented in this study can be found in online repositories. The names of the repository/repositories and accession number(s) can be found in the article/Supplementary material.

## Author contributions

X-XG: Conceptualization, Investigation, Supervision, Writing – original draft. Y-HZ: Writing – original draft, Writing – review & editing. H-MC: Writing – original draft, Data curation. NZ: Data curation, Writing – original draft. YH: Data curation, Writing – original draft. HL: Methodology, Writing – original draft. Z-YX: Conceptualization, Funding acquisition, Resources, Supervision, Writing – review & editing.

## Funding

The author(s) declare financial support was received for the research, authorship, and/or publication of this article. This work was financially supported by the National Natural Science Foundation of China (no. 32260927 and no. 32060835), Hainan Province Science and Technology Special Fund (ZDYF2022XDNY349), and Natural Science Foundation of Hainan Province (2019RC106).

## Conflict of interest

The authors declare that the research was conducted in the absence of any commercial or financial relationships that could be construed as a potential conflict of interest.

## Publisher’s note

All claims expressed in this article are solely those of the authors and do not necessarily represent those of their affiliated organizations, or those of the publisher, the editors and the reviewers. Any product that may be evaluated in this article, or claim that may be made by its manufacturer, is not guaranteed or endorsed by the publisher.

## Footnotes

^1^ http://smart.embl-heidelberg.de/

^2^ https://www.ncbi.nlm.nih.gov/Structure/cdd/cdd.shtml

^3^ https://www.genome.jp/tools-bin/clustalw

^4^ https://espript.ibcp.fr/ESPript/cgi-bin/ESPript.cgi

^5^ https://weblogo.threeplusone.com/create.cgi
