# Supplementary material for: Bioinformatic and functional characterization of cyclic-di-GMP metabolic proteins in Vibrio alginolyticus unveils key diguanylate cyclases controlling multiple biofilm-associated phenotypes
Source: Front Microbiol. 2023 Sep 21;14:1258415. doi: 10.3389/fmicb.2023.1258415 (PMC10552763; doi:10.3389/fmicb.2023.1258415)
Supplement: Supplementary file 1 [file Data_Sheet_1.pdf]

Supplementary Material

Content:

Supplementary Table S1.

Supplementary Table S2.

Supplementary Table S3.

Supplementary Figure S1.

Supplementary Figure S2.

Supplementary Figure S3.

Supplementary Figure S4.

Supplementary Figure S5.

Supplementary Figure S6.

Supplementary Figure S7.

Supplementary Figure S8.

Supplementary Figure S9.

Table S1. Strains and plasmids used in this work

Strain or Plasmid	Genotype	Source
HN08155(WT)	HN08155, wild-type, Amp^R	Laboratory collection
DH5α	F^- φ80 lac ZΔM15 Δ(*lacZYA-argF*) U169 *endA*1 *recA*1 *hsdR*17(rk-, mk+) supE44λ-thi-1 *gyrA*96 *relA*1 *phoA*	Weidi Biotechnology, Shanghai, China
TOP10	F^- *mcrA* Δ(mrr-*hsdR*MS-*mcrBC*) φ80 *lacZ*ΔM15 Δ*lacX*74 *recA*1 araΔ139 Δ(ara-leu)7697 *galU galK rpsL* (StrR) *endA*1 *nupG*	Weidi Biotechnology, Shanghai, China
β2163	Host are required for *pir* gene, DAP auxotroph	Laboratory collection
Δ*VA3903* (*cdgA*)	HN08155, in-frame deletion in *VA3903* gene, Amp^R	This work
Δ*VA4342*	HN08155, in-frame deletion in *VA4342* gene, Amp^R	This work
Δ*VA4622* (*pleD*)	HN08155, in-frame deletion in *VA4622* gene, Amp^R	This work
Δ*VA1591* (*cdgM*)	HN08155, in-frame deletion in *VA1591* gene, Amp^R	This work
Δ*VA2058* (*cdgK*)	HN08155, in-frame deletion in *VA2058* gene, Amp^R	This work
Δ*VA4033* (*dgcB*)	HN08155, in-frame deletion in *VA4033* gene, Amp^R	This work
Δ*VA3192*	HN08155, in-frame deletion in *VA3192* gene, Amp^R	This work
Δ*VA4663*	HN08155, in-frame deletion in *VA4663* gene, Amp^R	This work
Δ*VA2417* (*rocS*)	HN08155, in-frame deletion in *VA2417* gene, Amp^R	This work
Δ*VA3005* (*cdgJ*)	HN08155, in-frame deletion in *VA3005* gene, Amp^R	This work
Δ*VA4672* (*cdgC*)	HN08155, in-frame deletion in *VA4672* gene, Amp^R	This work
Δ*VA3251* (*mbaA*)	HN08155, in-frame deletion in *VA3251* gene, Amp^R	This work
Δ*VA3193*	HN08155, in-frame deletion in *VA3193* gene, Amp^R	This work
Δ*VA3814* (*lapD*)	HN08155, in-frame deletion in *VA3814* gene, Amp^R	This work
Δ*VA2701* (*csrD*)	HN08155, in-frame deletion in *VA2701* gene, Amp^R	This work
Δ*VA0068*	HN08155, in-frame deletion in *VA0068* gene, Amp^R	This work
Δ*VA4700*	HN08155, in-frame deletion in *VA4700* gene, Amp^R	This work
Δ*VA0356*	HN08155, in-frame deletion in *VA0356* gene, Amp^R	This work
Δ*VA0663*	HN08155, in-frame deletion in *VA0663* gene, Amp^R	This work
Δ*VA4408*	HN08155, in-frame deletion in *VA4408* gene, Amp^R	This work
Δ*VA0088*	HN08155, in-frame deletion in *VA0088* gene, Amp^R	This work
Δ*VA0108*	HN08155, in-frame deletion in *VA0108* gene, Amp^R	This work
Δ*cdgH*	HN08155, in-frame deletion in *cdgH* gene, Amp^R	Laboratory collection
Δ*flhF*	HN08155, in-frame deletion in *flhF* gene, Amp^R	Laboratory collection
Δ*VA3903*p3903	Overexpression of VA3903 in Δ*3903 mutant*, Amp^R, Cm^R	This work
Δ*VA4342*p4342	Overexpression of VA4342 in Δ*4342 mutant*, Amp^R, Cm^R	This work
Δ*VA4622*p4622	Overexpression of VA4622 in Δ*4622 mutant*, Amp^R, Cm^R	This work
Δ*VA1591*p1591	Overexpression of VA1591 in Δ*1591 mutant*, Amp^R, Cm^R	This work
Δ*VA2058*p2058	Overexpression of VA2058 in Δ*2058 mutant*, Amp^R, Cm^R	This work
Δ*VA4033*p4033	Overexpression of VA4033 in Δ*4033 mutant*, Amp^R, Cm^R	This work
Δ*VA3192*p3192	Overexpression of VA3192 in Δ*3192 mutant*, Amp^R, Cm^R	This work
Δ*VA4663*p4663	Overexpression of VA4663 in Δ*4663 mutant*, Amp^R, Cm^R	This work
Δ*VA2417*p2417	Overexpression of VA2417 in Δ*2417 mutant*, Amp^R, Cm^R	This work
Δ*VA3005*p3005	Overexpression of VA3005 in Δ*3005 mutant*, Amp^R, Cm^R	This work
Δ*VA4672*p4672	Overexpression of VA4672 in Δ*4672 mutant*, Amp^R, Cm^R	This work
Δ*VA3251*p3251	Overexpression of VA3251 in Δ*3251 mutant*, Amp^R, Cm^R	This work

Δ*VA3193*p3193	Overexpression of VA3193 in Δ*3193 mutant*, Amp^R, Cm^R	This work
Δ*VA3814*p3814	Overexpression of VA3814 in Δ*3814 mutant*, Amp^R, Cm^R	This work
Δ*VA2701*p2701	Overexpression of VA2701 in Δ*2701 mutant*, Amp^R, Cm^R	This work
Δ*VA0068*p0068	Overexpression of VA0068 in Δ*0068 mutant*, Amp^R, Cm^R	This work
Δ*VA4700*p4700	Overexpression of VA4700 in Δ*4700 mutant*, Amp^R, Cm^R	This work
Δ*VA0356*p0356	Overexpression of VA0356 in Δ*0356 mutant*, Amp^R, Cm^R	This work
Δ*VA0663*p0663	Overexpression of VA0663 in Δ*0663 mutant*, Amp^R, Cm^R	This work
Δ*VA4408*p4408	Overexpression of VA4408 in Δ*4408 mutant*, Amp^R, Cm^R	This work
Δ*VA0088*p0088	Overexpression of VA0088 in Δ*0088 mutant*, Amp^R, Cm^R	This work
Δ*VA0108*p0108	Overexpression of VA0108 in Δ*0108 mutant*, Amp^R, Cm^R	This work
pDM4	Suicide vector, required *pir* gene, Cm^R	Laboratory collection
pBAD/Myc-HisA-Cm	araBAD promoter (PBAD), Cm^R	Laboratory collection

Table S2. Primers used in this work

Primer	Sequence (5'-3')
**Knockout of 22 c-di-GMP metabolic genes**	
VA3903-UF-*Bgl*II	GAAGATCTAATCTCATCCGGTTGGCTCT
VA3903-UR	CGTTGAAATTTCATTTTGTACAAAGAAGTGGCAAACACCA
VA3903-DF	TGGTGTTTGCCACTTCTTTGTACAAAATGAAATTTCAACG
VA3903-DR-*Xho*I	CCCTCGAGGCAAGTCAGTGAGCATTCGT
VA3903-TF	CAATGTGAGGATGAAACCGG
VA3903-TR	GATTACCTTAATGACCTCGA
VA1591-UF-*Bgl*II	GAAGATCTGCGCCTACTTCTTCATCAGAG
VA1591-UR	TCGCCAATCGACACATTATTTCGTTTGATAACGCTCTTG
VA1591-DF	CAAGAGCGTTATCAAACGAAATAATGTGTCGATTGGCGA
VA1591-DR-*Xho*I	CCCTCGAGTACGCATCCACATAACCAGAA
VA1591-TF	GTAATCGTTCAATACTTTCTG
VA1591-TR	ACGACAATGGTTGAATGACG
VA2058-UF-*Sal*I	GCGTCGACGTGAAGTCGGACTAGAGGGAG
VA2058-UR	GCGCTTAAATCTTCGTAGCAAAATGAGAGGCTAAATCGCC
VA2058-DF	GGCGATTTAGCCTCTCATTTTGCTACGAAGATTTAAGCGC
VA2058-DR-*Xba*I	GCTCTAGACAATCTGCTGGTGCAAATGA
VA2058-TF	TCAGCTATCGCAGCTAAATC
VA2058-TR	CTGTTCCAGAGGCTATTCCT
VA4622-UF-*Bgl*II	GAAGATCTCGCGGTATTCTTCTTTATCTTC
VA4622-UR	ACCACCGTATGTGTATTTCTATTCGCATTGATGTCCCTGT
VA4622-DF	ACAGGGACATCAATGCGAATAGAAATACACATACGGTGGT
VA4622-DR-*Xho*I	CCCTCGAGGGTCATTCAGTGCATTACTACGAT
VA4622-TF	GCAGTTGTGAAGCACATTATC
VA4622-TR	TTAGGTATACACCTTCTAGA
VA4342-UF-*Bgl*II	GAAGATCTGGTTCGACCATTAAGGAGGAT
VA4342-UR	ACTTGACGAAGCTCAGGTTGAGACTCTGACTTGTGCGCAT
VA4342-DF	ATGCGCACAAGTCAGAGTCTCAACCTGAGCTTCGTCAAGT
VA4342-DR-*Xho*I	CCCTCGAGGCAAGTGCATCAACTACCCA
VA4342-TF	TAAATGACGGTGATGTGAAG
VA4342-TR	AACTCGACTTTGCGCTGTAA
VA3192-UF-*Bgl*II	GAAGATCTTTATTCAACAATGCTCGGAG
VA3192-UR	TGTTGTCCTCAAACACGCTG CATAACACTTTATGTTCCCA
VA3192-DF	TGGGAACATAAAGTGTTATGCAGCGTGTTTGAGGACAACA
VA3192-DR-*Xho*I	CCCTCGAGTTGAAGTAATGAAGCTGGGC
VA3192-TF	CAATCAGTGCTGATTAATAC
VA3192-TR	TCAAGCATAATAAATGGCAG
VA4033-UF-*Bgl*II	GAAGATCTCTTGATAGGGCGTCAGTTCA
VA4033-UR	GGCATCACGCGGTTACGACCTTTCAAATTAGCGGTCGATT
VA4033-DF	AATCGACCGCTAATTTGAAAGGTCGTAACCGCGTGATGCC
VA4033-DR-*Spe*I	CGGACTAGTGCAGAGCGAGAAGCAAGTAA
VA4033-TF	CCGAACCAGCAAACGCATCA

VA4033-TR	AGTGCGAGGATATGACGATG
VA4663-UF-*Bgl*II	GAAGATCTACCCGTCATGTTTATACTGC
VA4663-UR	ACTGTCTCCTGCAATTCGCA TGGCCAAATAAGCTTTGCTC
VA4663-DF	GAGCAAAGCTTATTTGGCCATGCGAATTGCAGGAGACAGT
VA4663-DR-*Xho*I	CCCTCGAGGTGTGATGCTCCAATCTGGA
VA4663-TF	GCTGATATCTGTGTATTGAG
VA4663-TR	CCCACTGCAACTTGAGAAAC
VA2417-UF-*Bgl*II	GAAGATCTAATCGCAACTTTTAACCACTTG
VA2417-UR	TGACGACGATTCTGTACAAATAGCGTGGCAAACCAATGTT
VA2417-DF	AACATTGGTTTGCCACGCTATTTGTACAGAATCGTCGTCA
VA2417-DR-*Xho*I	CCCTCGAGATAAGACGCGAGGCAGCAC
VA2417-TF	GTCTTGATAGCTGATGTACC
VA2417-TR	TCTAGCTCGACATCGCTAGT
VA4672-UF-*Bgl*II	GAAGATCTAACTTTGACGGCGGTGCTTA
VA4672-UR	GTAGGCGTATTGTCCGCCTGCTCAGGGATTTCATAATCTA
VA4672-DF	TAGATTATGAAATCCCTGAGCAGGCGGACAATACGCCTAC
VA4672-DR-*Xho*I	CCCTCGAGCAACTTGGCAATGCTCAATC
VA4672-TF	CTATCTATCTCGATGGTACG
VA4672-TR	ATTGGCTTCCAACTGTGCAC
VA3005-UF-*Bgl*II	GAAGATCTACGGTGAACTCAATCAGCAA
VA3005-UR	ACGTTTTGACTCCAGCGAACGCGAGCAACATACGTTGTAT
VA3005-DF	ATACAACGTATGTTGCTCGCGTTCGCTGGAGTCAAAACGT
VA3005-DR-*Xho*I	CCCTCGAGGAATGCCGCTCGCAGTGCTA
VA3005-TF	TACGCGATGTATCATCGAAG
VA3005-TR	TCACCTGCGACATCGTTGAT
VA3251-UF-*Bgl*II	GAAGATCTCTTGGTACGCCATGCGTTAG
VA3251-UR	TAAGATGTCCCTTGAACAGTGATGACTCGATTGCTTAACT
VA3251-DF	AGTTAAGCAATCGAGTCATCACTGTTCAAGGGACATCTTA
VA3251-DR-*Xho*I	CCCTCGAGTCGATAAACTTCGGAATAAA
VA3251-TF	TGCGACTCAGATAGAATCAC
VA3251-TR	ACTAACTGTTTGAAACTCAA
VA2701-UF-*Bgl*II	GAAGATCTAAGGCCAACTGCAAACTCGT
VA2701-UR	TTACGCCAGCGATTTCGTCTAAACATGGCGCAGATGACGA
VA2701-DF	TCGTCATCTGCGCCATGTTTAGACGAAATCGCTGGCGTAA
VA2701-DR-*Xho*I	CCCTCGAGACACTGCGAATCGAACGATG
VA2701-TF	GTGATAAAGCGACTGGCGAA
VA2701-TR	CGAACGTTGCAGTTCTAACG
VA3193-UF-*Bgl*II	GAAGATCTTGAGCTTCGAGCAGCATAGT
VA3193-UR	GTGATTACCTCTAACGGCAT CACCATCACATTAAGCTGTG
VA3193-DF	CACAGCTTAATGTGATGGTGATGCCGTTAGAGGTAATCAC
VA3193-DR-*Xho*I	CCCTCGAGTAGAGCTGGGATTGGTCGTT
VA3193-TF	TACGTGCTTTGTGCATGTGG
VA3193-TR	GGCTTAGAGATAAGAACTCG
lapD-UF-*Bgl*II	GAAGATCTAGAGACACATGAACAGCTCG

lapD-UR	TCGACGATGAATCCTTGGAACATTCCCACTACAAGCTTCT
lapD-DF	AGAAGCTTGTAGTGGGAATGTTCCAAGGATTCATCGTCGA
lapD-DR-*Spe*I	CGGACTAGTGTTGATGAGAAGTGAGGCGA
lapD-TF	CGAGTATTGAGCGATAAGTG
lapD-TR	CCCATCACTTTGCTGAACAG
VA0068-UF-*Bgl*II	GAAGATCTTCACACTGCGGTTAATTGGG
VA0068-UR	TCAGAAAGCGGTTGAGGATG TAGCGCGACAATAACCAGAA
VA0068-DF	TTCTGGTTATTGTCGCGCTACATCCTCAACCGCTTTCTGA
VA0068-DR-*Xho*I	CCCTCGAGAGGCATGGATCAGCGACATA
VA0068-TF	TTGATGCGCTATTGCGCCAT
VA0068-TR	GATACATAGGGTCGAGATAC
VA4700-UF-*Bgl*II	GAAGATCTAATAGGCACGGTTTGGGTCC
VA4700-UR	GCCTCTTCATAAACGTCTAAGATGCAACTAAGGAGTTGCG
VA4700-DF	CGCAACTCCTTAGTTGCATCTTAGACGTTTATGAAGAGGC
VA4700-DR-*Xho*I	CCCTCGAGTACTTGATTGGCTCGCGCAA
VA4700-TF	ACTTTCCGTCACTTCGGATA
VA4700-TR	AAGTGTCACCAGCAAACACG
VA0356-UF-*Bgl*II	GAAGATCTGCTATTGGAAAACCAACCGGG
VA0356-UR	CTATGACATAGCGATACGCA TGTGCCGACTCGCTTAGGTA
VA0356-DF	TACCTAAGCGAGTCGGCACATGCGTATCGCTATGTCATAG
VA0356-DR-*Xho*I	CCCTCGAG GTAACGATGAGTTGAATGAC
VA0356-TF	ACCGAACACGTATACCCGAT
VA0356-TR	TACAACGTAGGAGCGTCACC
VA0108-UF-*Bgl*II	GAAGATCTGCATTGCGTGAACGTAAGCA
VA0108-UR	TGTGTTGGCCACTCTTCTAG CGTAGCAGTAGACATTGCCT
VA0108-DF	AGGCAATGTCTACTGCTACGCTAGAAGAGTGGCCAACACA
VA0108-DR-*Xho*I	CCCTCGAG TGCCGATCAGGTTGGCTTTC
VA0108-TF	TCAGCGTCGACGTAAGGATT
VA0108-TR	GCCAAGTCGACAGCAACGAT
VA0663-UF-*Bgl*II	GAAGATCTCATGTACGTCCACAACGGTT
VA0663-UR	CAGTTAAGCTGATTTGTTCCGGATTCCAAGATTCCCATGA
VA0663-DF	TCATGGGAATCTTGGAATCCGGAACAAATCAGCTTAACTG
VA0663-DR-*Spe*I	CGACTAGTACAGATGCTCTTGGATACCT
VA0663-TF	CGTTAAGCTGTCTCGCTCAT
VA0663-TR	AACACGAGGTTAGTGATGTG
VA4408-UF-*Bgl*II	GAAGATCTATGCTGTGCGGTCTCATAGC
VA4408-UR	TCATAATGACTTCGCTGAGCAAGCGCCTTATTCAGCATAT
VA4408-DF	ATATGCTGAATAAGGCGCTTGCTCAGCGAAGTCATTATGA
VA4408-DR-*Xho*I	CCCTCGAGGGTCAGCATTGGCAGATCAA
VA4408-TF	GCAGCGCCTGCATACTTTCT
VA4408-TR	CAGCATCAGCACTCGACACA
VA0088-UF-*Bgl*II	GAAGATCTCTTGCGCTGCTATCAACGCT
VA0088-UR	CACTTACAGGTCGGCTAAA CGAAGGAATAGCGAAGCGAA
VA0088-DF	TTCGCTTCGCTATTCCTTCGTTTAGCCGACCTGTAAGTG

VA0088-DR-*Xho*I	CCCTCGAGGTCAGTGTCGTACTTGCATG
VA0088-TF	CTGGCTGGCTTGCGGTTTTA
VA0088-TR	TTTGGTGAAGACACTCGCGAC
**Complementation for 22 c-di-GMP metabolic genes**	
pBAD(Cm)-RF	CCATATGGGAATTCGAAGCT
pBAD(Cm)RR	TCGAGCTCGGATCCATGGTTA
pBAD-TF	CGTCACACTTTGCTATGCCA
pBAD-TR	AATCTTCTCTCATCCGCCAA
pBAD-3903-F	TAACCATGGATCCGAGCTCGAATGTTGCTTGTGGTGTTTGC
pBAD-3903-R	AGCTTCGAATTCCCATATGGACGTTGAAATTTCATTTTGT
pBAD-1591-F	TAACCATGGATCCGAGCTCGAATGACACAAAGCACCGCTCA
pBAD-1591-R	AGCTTCGAATTCCCATATGGATCGCCAATCGACACATTAT
pBAD-2058-F	TAACCATGGATCCGAGCTCGAATGGTAAACCAAAGGCGATT
pBAD-2058-R	AGCTTCGAATTCCCATATGGAGCGCTTAAATCTTCGTAGC
pBAD-4622-F	TAACCATGGATCCGAGCTCGAATGCAACCGACAGGGACATC
pBAD-4622-R	AGCTTCGAATTCCCATATGGACTTGCATTTACCACCGTAT
pBAD-4342-F	TAACCATGGATCCGAGCTCGAATGACGTATGCGCACAAGTC
pBAD-4342-R	AGCTTCGAATTCCCATATGGAGGGACTTGACGAAGCTCAG
pBAD-3192-F	TAACCATGGATCCGAGCTCGAATGAATGTAAGAAGTTTTGT
pBAD-3192-R	AGCTTCGAATTCCCATATGGAACACGCTGTTTTTTCATTTC
pBAD-4033-F	TAACCATGGATCCGAGCTCGAATGACCGACGAGTTTAAAAA
pBAD-4033-R	AGCTTCGAATTCCCATATGGAATGGGCATCACGCGGTTAC
pBAD-4663-F	TAACCATGGATCCGAGCTCGAATGTCATTACGAGCAAAGCT
pBAD-4663-R	AGCTTCGAATTCCCATATGGAACTGTCTCCTGCAATTCGC
pBAD-2417-F	TAACCATGGATCCGAGCTCGAATGCCTTCCCAGCAATTAAA
pBAD-2417-R	AGCTTCGAATTCCCATATGGGTTTTTGTGACGACGATTCT
pBAD-4672-F	TAACCATGGATCCGAGCTCGAATGGGGAGATTGCAAACATT
pBAD-4672-R	AGCTTCGAATTCCCATATGGGGAAGAGTAGGCGTATTGTC
pBAD-3005-F	TAACCATGGATCCGAGCTCGAATGAATACAACGTATGTTGC
pBAD-3005-R	AGCTTCGAATTCCCATATGGCGCAGTTGCGACGTTTTGAC
pBAD-3251-F	TAACCATGGATCCGAGCTCGAATGAAGTTAAGCAATCGAGT
pBAD-3251-R	AGCTTCGAATTCCCATATGGATAAGATGTCCCTTGAACAG
pBAD-2701-F	TAACCATGGATCCGAGCTCGAATGATCGTCATCTGCGCCAT
pBAD-2701-F	AGCTTCGAATTCCCATATGGTTTTTTACGCCAGCGATTTCG
pBAD-3193-F	TAACCATGGATCCGAGCTCGAATGAATACACAGCTTAATGTG
pBAD-3193-R	AGCTTCGAATTCCCATATGGCTTCAAAATAGTGATTACCT
pBAD-3814-F	TAACCATGGATCCGAGCTCGAATGACCCTATATAAGAAGCT
pBAD-3814-R	AGCTTCGAATTCCCATATGGTTCCTTGTCGACGATGAATC
pBAD-0068-F	TAACCATGGATCCGAGCTCGAGTGCAGCGTTTTCTGGTTAT
pBAD-0068-R	AGCTTCGAATTCCCATATGGCTCAGCCGTCAGTCGCTCAA
pBAD-4700-F	TAACCATGGATCCGAGCTCGAATGATGAGCACCTCGCAACT
pBAD-4700-R	AGCTTCGAATTCCCATATGGGGCTGTTTTTGCCTCTTCAT
pBAD-0356-F	TAACCATGGATCCGAGCTCGAGTGAAAATACCTAAGCGAGT
pBAD-0356-R	AGCTTCGAATTCCCATATGGAGCAATATCATCGAAACTAT

pBAD-0108-F	TAACCATGGATCCGAGCTCGAATGTCTACTGCTACGACTCA
pBAD-0108-R	AGCTTCGAATTCCCATATGGCTTCATGTTAACCAAATGTG
pBAD-0663-F	TAACCATGGATCCGAGCTCGAATGGGAATCTTGGAATCCGA
pBAD-0663-R	AGCTTCGAATTCCCATATGGGCTCCAGTTAAGCTGATTTG
pBAD-4408-F	TAACCATGGATCCGAGCTCGAATGCTGAATAAGGCGCTTTT
pBAD-4408-R	AGCTTCGAATTCCCATATGGCCAATCATAATGACTTCGCT
pBAD-0088-F	TAACCATGGATCCGAGCTCGAATGTTTCGCTTCGCTATTCC
pBAD-0088-R	AGCTTCGAATTCCCATATGGACGAAAAAGTTTTTCCGCAC
**qPCR assay**	
vpsQ-qF	CCTACTTAGGTGGATCGAGTACGCT
vpsQ-qR	ATGCCTATCCAGTTGGTGCTGAG
VA4199-qF	TCACCGAGATATGGCAGTCACGT
VA4199-qR	CCAGTTCTTGACGCATCACATCGA
VA4200-qF	GATCTGCACGCTCGTTGCCTTAT
VA4200-qR	CAAAGCGACGAGCATCAAAAGGTG
vpsN-qF	GCGATACCATTTCCGTGCAGGTC
vpsN-qR	GCTTTGATTCGGCCAAGGTAGGG
vpsM-qF	CGCAACGACTTCGACGATCATACC
vpsM-qR	CTGCCTTTTGCACCGTTAGAGCC
wcaJ-qF	GCCTTGATCTCTCCAGTCCTGCTC
wcaJ-qR	CGTGAGGGCCTTGATCCATAGTG
flgB-qF	GACAATGCACTAGGCATCCACCAG
flgB-qR	CCGACCATCCGTACGACTAAGACC
flgF-qF	CGATCACACGGGTAAAGAAGGGTT
flgF-qR	GGAAGCACAGAGATGGTGCCATCC
flgG-qF	ACGGCTTCTTCCAAGTGACGTTG
flgG-qR	CTTCACCATCCGTACCGACAGTG
flgHI-qF	GCAAATGGCAACCTCGTTATCCGT
flgHI-qR	CGGTGCCAGAGTACTGAATTCTTGC
flgIII-qF	CTAGAGTTCGACCCTGCAGATGGC
FlgIII-qR	GCCGTTAGGTTGGCTAACATTCAGG
flgK-qF	TCACCGCTAACTTGGTGCCTTCG
flgK-qR	GCTCTAAGCGAGCGACAGAAGC
flaB-qF	TCAACCTAACGGACAGCTACGGC
flaB-qR	CCTTCGACTTTGTTGTTGCCAGCA
fliA-qF	CTGAAGTAGCCACGCATCTCGG
fliA-qR	GGCAACCCCTTTGAATGGGTTACTG
flhA-qF	CCAGGTCAGGTGTATGGCATGAT
flhA-qR	GTCAATAACTGGCTCAGGTGCGTC
fliP-qF	TGACCATGCTTGGCTTCTTGCC
fliP-qR	ACAGCGCTATACCAATGATGACCTG
fliN-qF	CCGAAGATGAGCGTCGTAAACTCG
fliN-qR	TTCACGACCACGACTTCGCCATG
fliF-qF	TGCGTTGAGTAATCAGCCTCCAGC

fliF-qR	GCAACAGAAACGGTCTGACGAGC
fliE-qF	CGCCATGATGGTAGAAGCAACCAAT
fliE-qR	GGAAACATCGGCATCACCACGATC
fliS-qF	CATGCAAGCAGGCAACATTCCAG
fliS-qR	ACGTCGTCGATCAGTTGAGGATC
flaA-qF	GTGGCAAGAGCTACGCAGCAG
flaA-qR	GCTAGCTGCTCAATGTCGTCGC
flaB-qF	CGGACAGCTATGGTCAAGAGCAAG
flaB-qR	CCGTCTTGGTCTACTGAGGCTT
flaF-qF	GCTGGCGATGATATCGAAGAGCT
flaF-qR	GCTGTAACCCCAGTTCACTCGC
lafA-qF	TTGCAGCGACTCTTGCTGACG
lafA-qR	CTCAAGACGGTTGATGTTCGCACC
lafT-qF	CTACAGCGTACAGCAGAAGCACT
lafT-qR	GTACCTACGAGAGCTGCTGCAAC
gyrB-qF	CGTACATCAAACGTGGTGAGCGT
gyrB-qR	CTTGCTACTGGCATCGTACGCTC

Table S3. Transmembrane and sensory partner domains located in the GGDEF-, EAL-, and HD-GYP-containing proteins

Total c-di-GMP metabolic proteins (63)	GGDEF-only (32)	EAL-only (11)	HD-GYP (4)	Hybrid (16)
Transmembrane helices (TM) n (%)	19 (59)	3 (27)	0 (0)	9 (56)
Sensory partner domain n (%)	13 (40)	2 (18)	-	12 (75)
With PAS domain	6	1	-	5
With HAMP domain	1	-	-	4
With CHASE domain	2	-	-	-
With REC domain	1	1	-	-
With GAF domain	1	-	-	2
With PBPb domain	2	-	-	-
With TPR domain	1	-	-	-
With CBS domain	-	1	-	-
With FIST domain	-	-	-	1

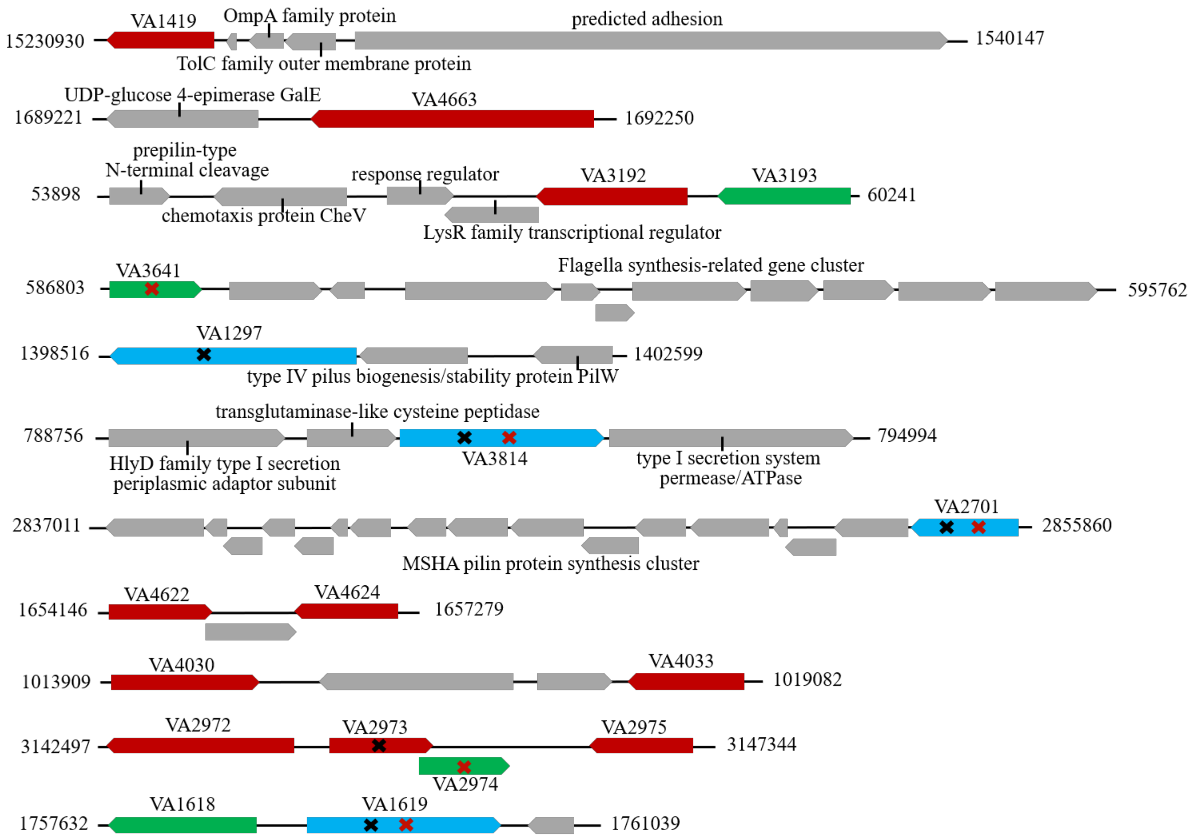

Supplementary Figure S1. Neighboring gene arrangements of several predicted genes encoding c-di-GMP metabolic proteins in the genome of *V. alginolyticus*. Red arrows represent genes encoding GGDEF domain proteins, green arrows represent genes encoding EAL domain proteins, blue arrows represent genes encoding hybrid domain proteins, grey arrows represent proteins encoded by other related functional genes. Black 'x' indicates that the GGDEF domain is intact (*e.g.*, GGDEF-GADEF), and red 'x' indicates the EAL domain. The size is not drawn to scale.

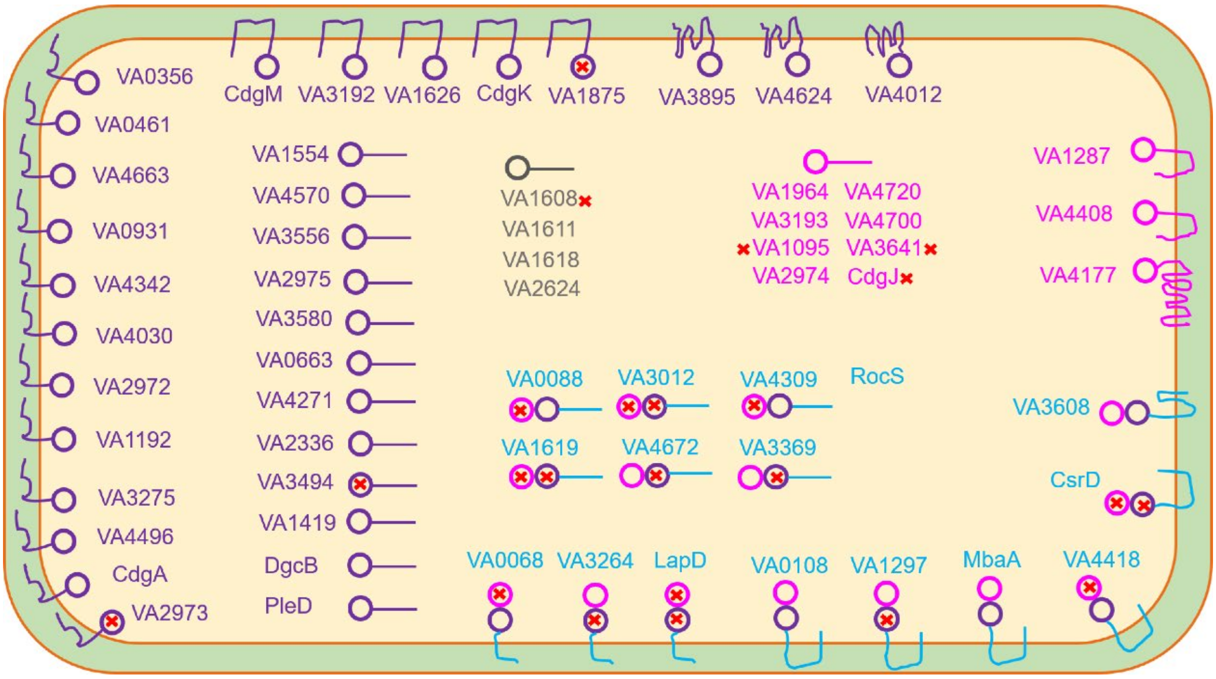

Supplementary Figure S2. Putative localization of 63 c-di-GMP metabolic proteins of *V. alginolyticus* strain HN08155. The rectangular shape represents a cell of *V. alginolyticus*, in that cytoplasm (yellow), periplasm (green regions), and inner and outer membranes (orange lines) are shown. The localization of c-di-GMP metabolic enzymes in the membrane or cytoplasm is predicted based on the amount of TM that they contain, the positions of all enzymes in the shape is arbitrary, 'x' indicates that the GGDEF or EAL domain is incomplete.

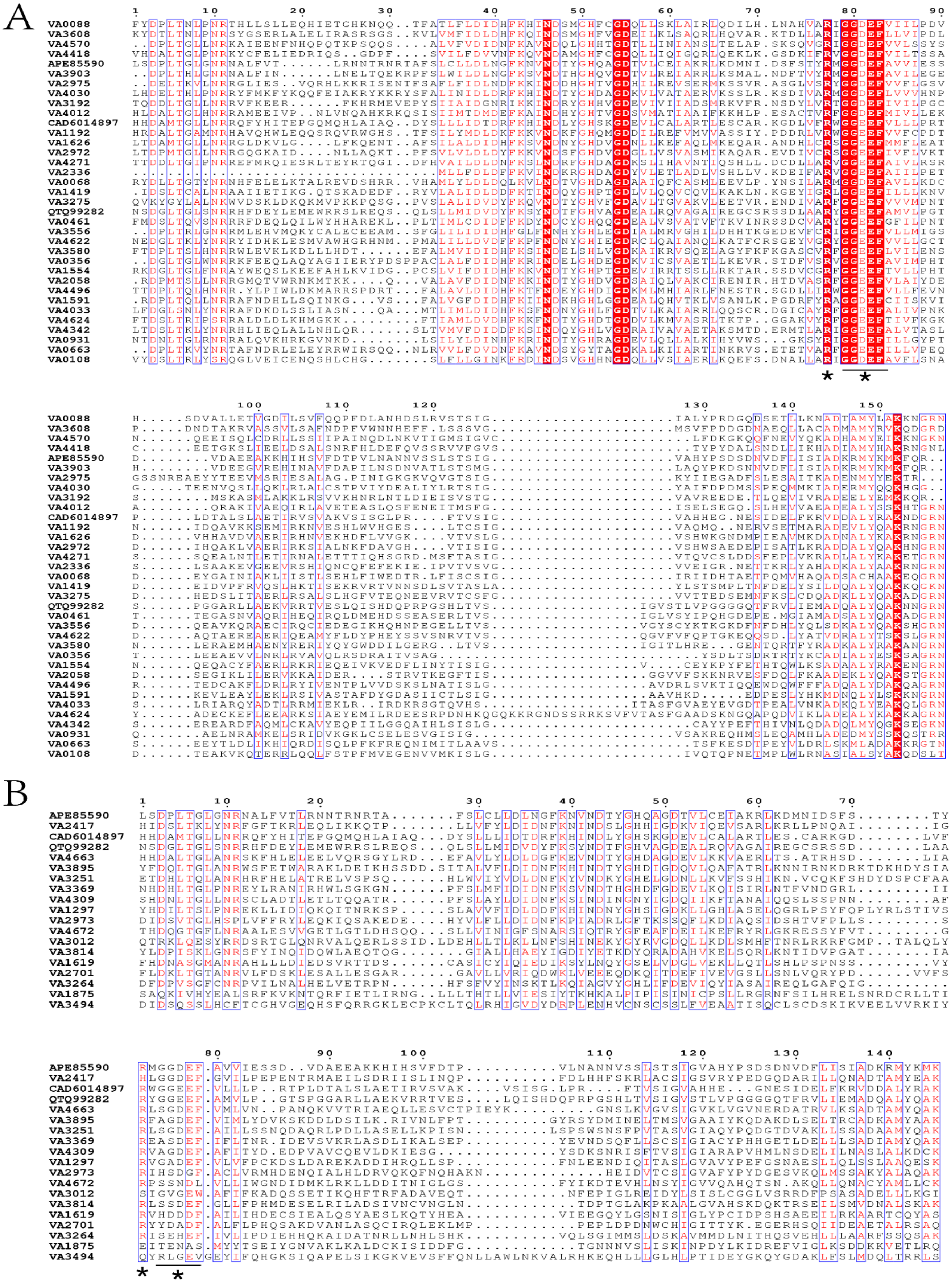
A
VA0088
VA3608
VA4570
VA4418
APE85590
VA3903
VA2975
VA4030
VA3192
VA4012
CAD6014897
VA1192
VA1626
VA2972
VA4271
VA2336
VA0068
VA1419
VA3275
QTQ99282
VA0461
VA3556
VA4622
VA3580
VA0356
VA1554
VA2058
VA4496
VA1591
VA4033
VA4624
VA4342
VA0931
VA0663
VA0108
B
APE85590
VA2417
CAD6014897
QTQ99282
VA4663
VA3895
VA3251
VA3369
VA4309
VA1297
VA2973
VA4672
VA3012
VA3814
VA1619
VA2701
VA3264
VA1875
VA3494

Supplementary Figure S3. (A) Sequence alignments of GGDEF domains from *V. alginolyticus* and other related species generated with ClustalW and formatted with ESPript 3.0. Key residues responsible for active site and allosteric inhibitory are marked with an '*'. The following sequences were used for sequence alignment: 27 proteins containing only the GGDEF domain and 5 proteins containing the bifunctional domain (only the GGDEF domain of these proteins was used for sequence alignment) in *V. alginolyticus*. The conserved GGD/EEF domains of all 32 proteins were predicted to be active. (B) Sequence alignments of GGDEF domains from the remaining 5 proteins containing only the GGDEF domain and 11 proteins containing the bifunctional domain. The conserved GGD/EEF domains of all 16 proteins were predicted to be inactive, but the S/AGDEF domains were shown to have some DGC activity. Three protein sequences of YdaM from *E. coli* (CAD6014897), WspR from *P. aeruginosa* (QTQ99282) and the transcriptional regulator CdgA (APE85590) from *V. parahaemolyticus* were used as references for BlastP analysis of GGD/EEF domains.

A

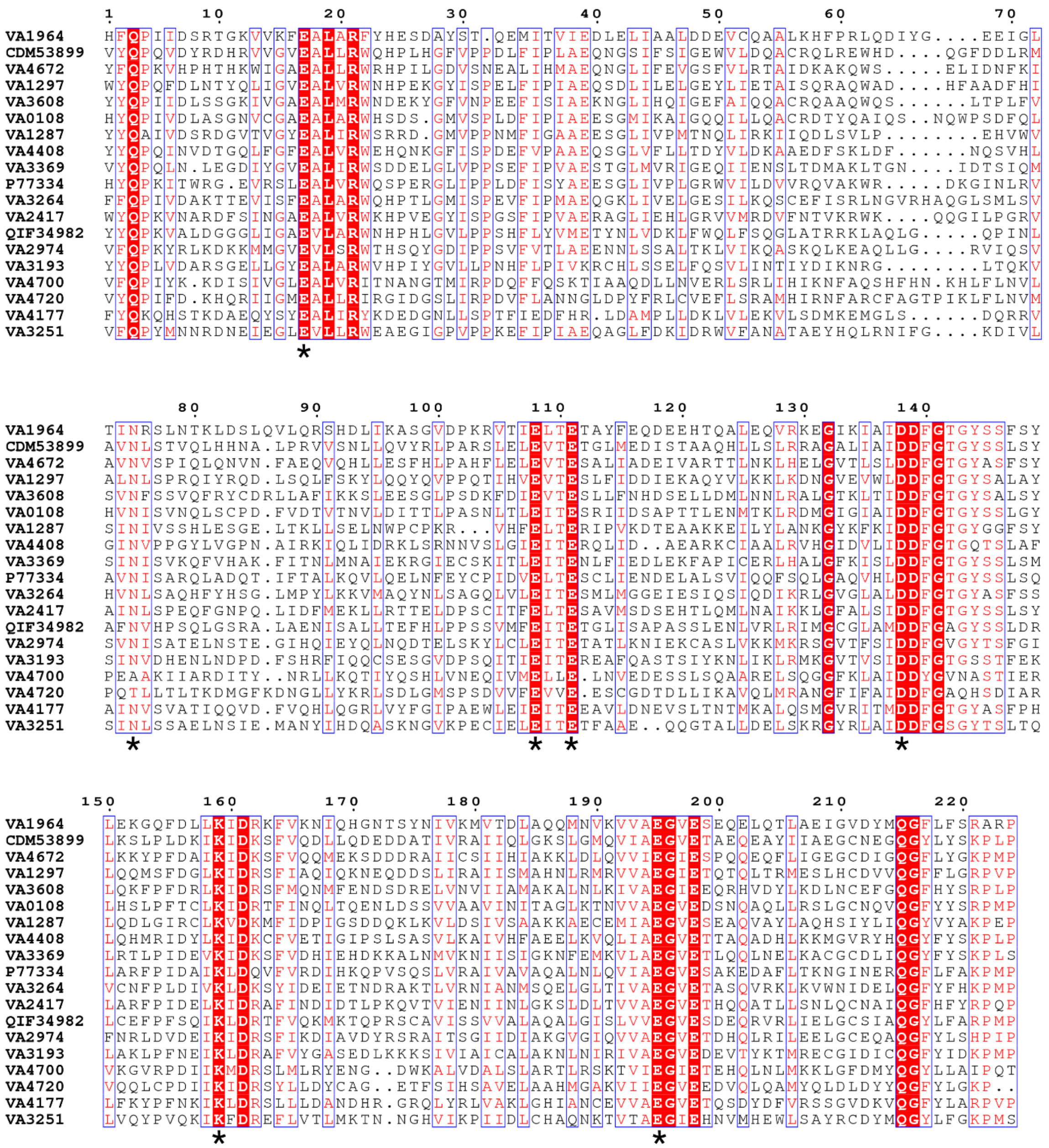

B

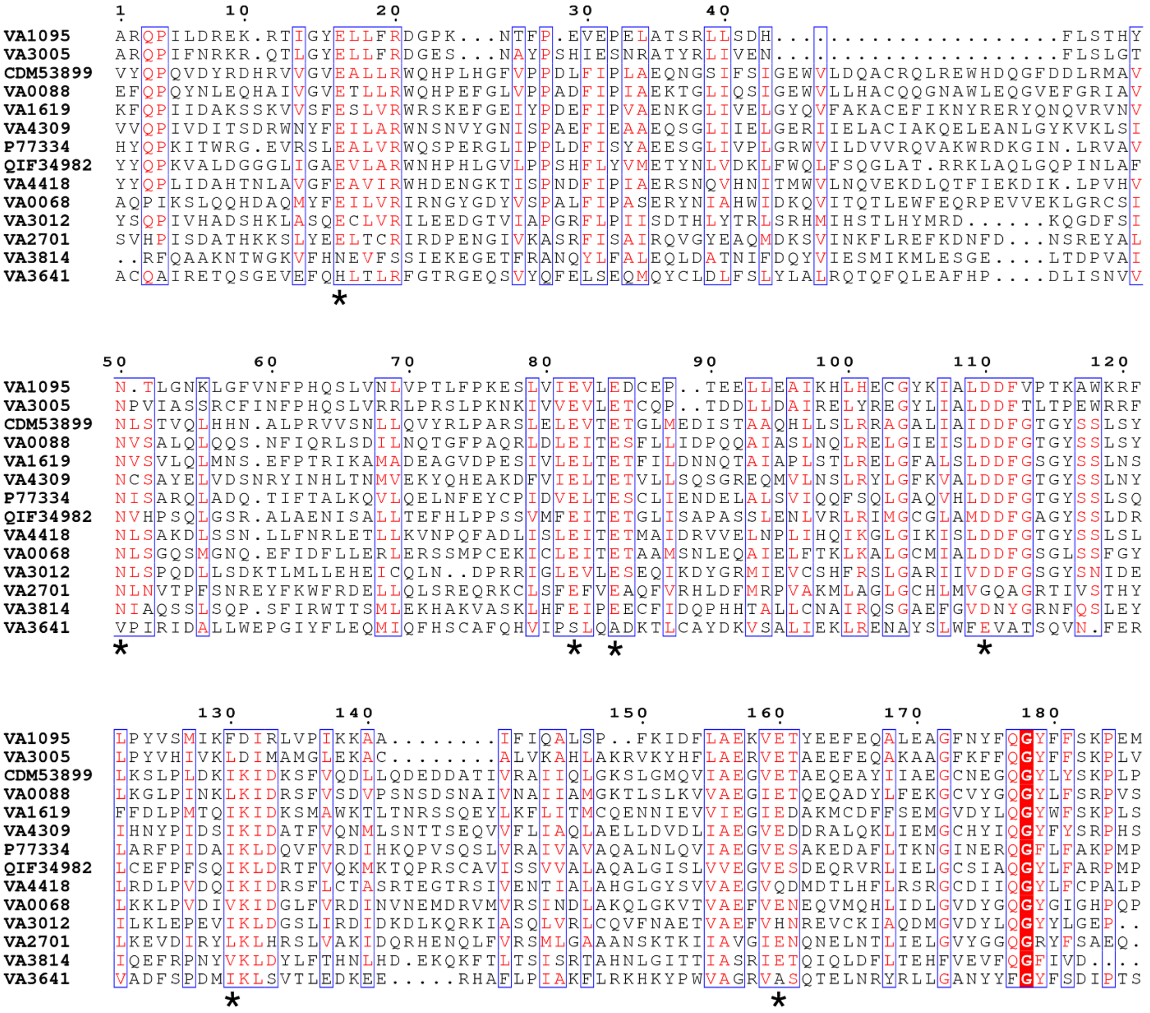

Supplementary Figure S4. (A) Sequence alignments of EAL domains from *V. alginolyticus* and other related species generated with ClustalW and ESPript 3.0. Key residues responsible for active site are marked with an '*'. The following sequences were used for sequence alignment: 8 proteins containing only the EAL domain and 8 proteins containing the bifunctional domain (only the EAL domain of these proteins were used for sequence alignment) in *V. alginolyticus*. The conserved EAL domains of all 16 proteins were predicted to be active. (B) Sequence alignments of EAL domains from the remaining 3 proteins containing only the EAL domain and 8 proteins containing the bifunctional domain. The conserved EAL domains of all 11 proteins were predicted to be inactive. Three protein sequences of BifA from *P. aeruginosa* WS394 (CDM53899), PdeR from *Escherichia coli* K-12 (P77334), and RocR from *P. aeruginosa* (QIF34982) were used as references for Blastp analysis of EAL domains.

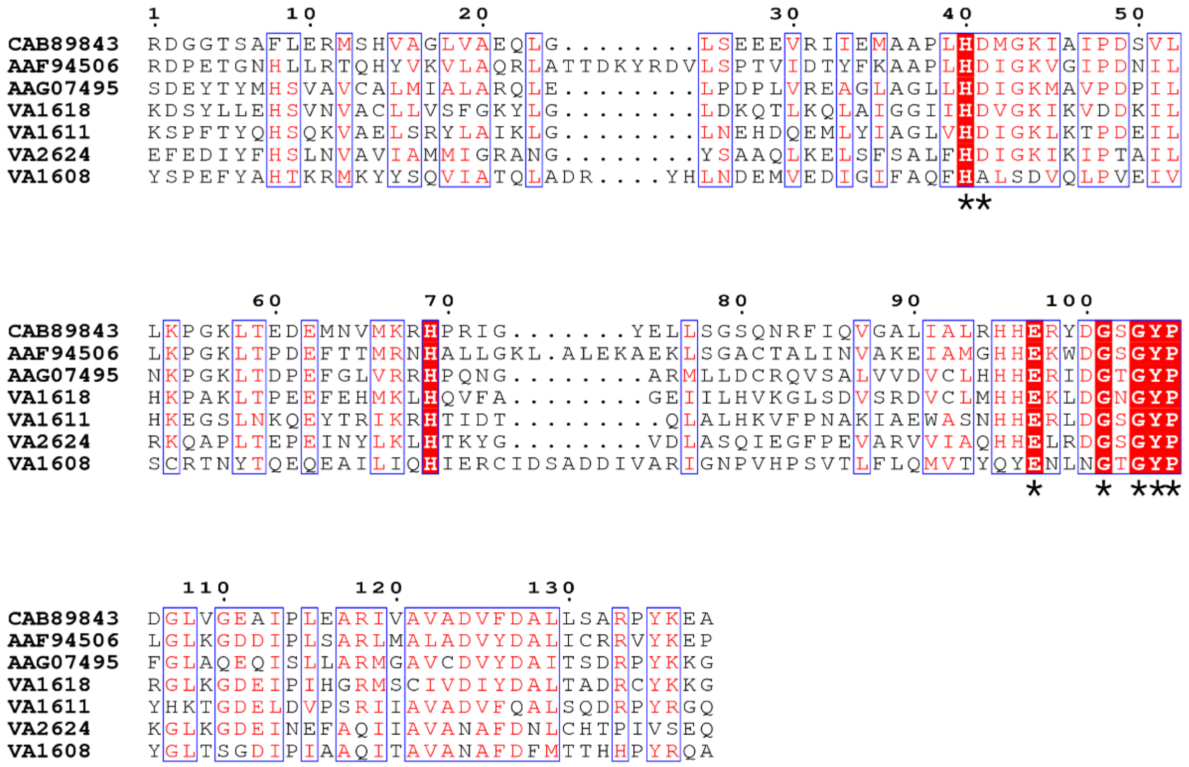

Supplementary Figure S5. Sequence alignments of HD-GYP domains from *V. alginolyticus* and other related species generated with ClustalW and ESPript 3.0. Key residues responsible for active site are marked with an '*'. The following sequences were used for sequence alignment: 4 proteins containing only the HD-GYP domain in *V. alginolyticus*. Three protein sequences from *X. campestris* (CAB89843), *P. aeruginosa* PAO1 (AAG07495), *V. cholerae* O1 biovar El Tor str. N16961 (AAF94506) were used as references for Blastp analysis of HD-GYP domains.

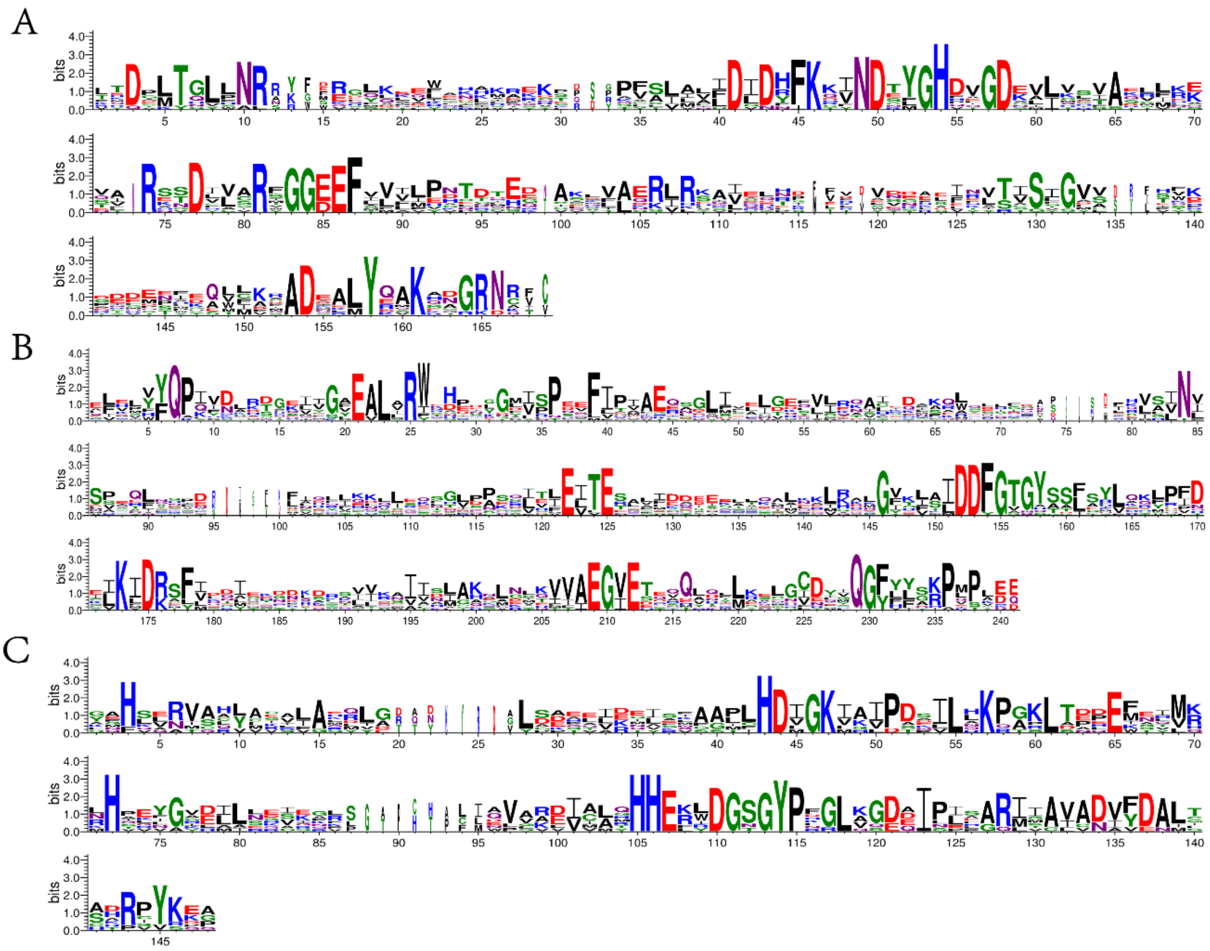

Supplementary Figure S6. Sequence logo of the GGDEF, EAL and HD-GYP domains completed using WebLogo 3: Create. (A) Sequence logo of the GGDEF domain derived from an alignment of 13 sequences (VA1554, VA0356, VA0461, VA4030, VA3192, VA1626, VA2058, VA2972, VA4496, VA3275, VA3608, CAD6014897, and QTQ99282). (B) Sequence logo of the EAL domain derived from an alignment of 11 sequences (VA2417, VA3264, VA0108, VA1297, VA3251, VA4672, VA3608, VA3369, CDM53899, P77334, and QIF34982). (C) Sequence logo of the HD-GYP domain derived from an alignment of 8 sequences (VA1618, VA2624, AAS97405, CAB89843, AAG07495, AAF95483, AAF96122, and AAF94506).

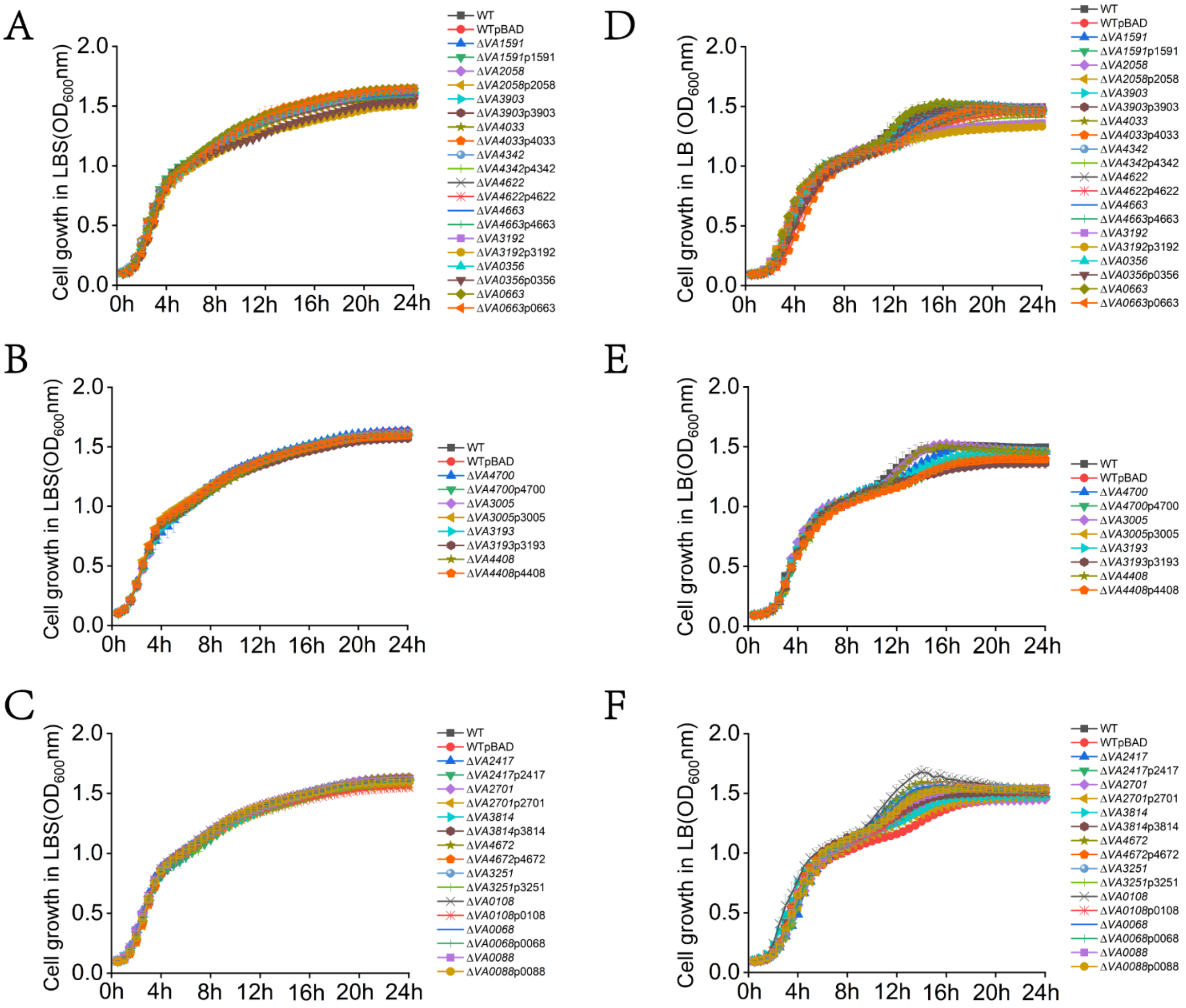

Supplementary Figure S7. Growth curves. Kinetic growth assays in LBS (A-C) and LB (D-F) medium for wild-type (WTpBAD) and 22 c-di-GMP metabolizing variants**.** OD_{600} readings were measured every 30 mins for 24 h in an EPOCH2 microplate reader. Two biological replicates were implemented.

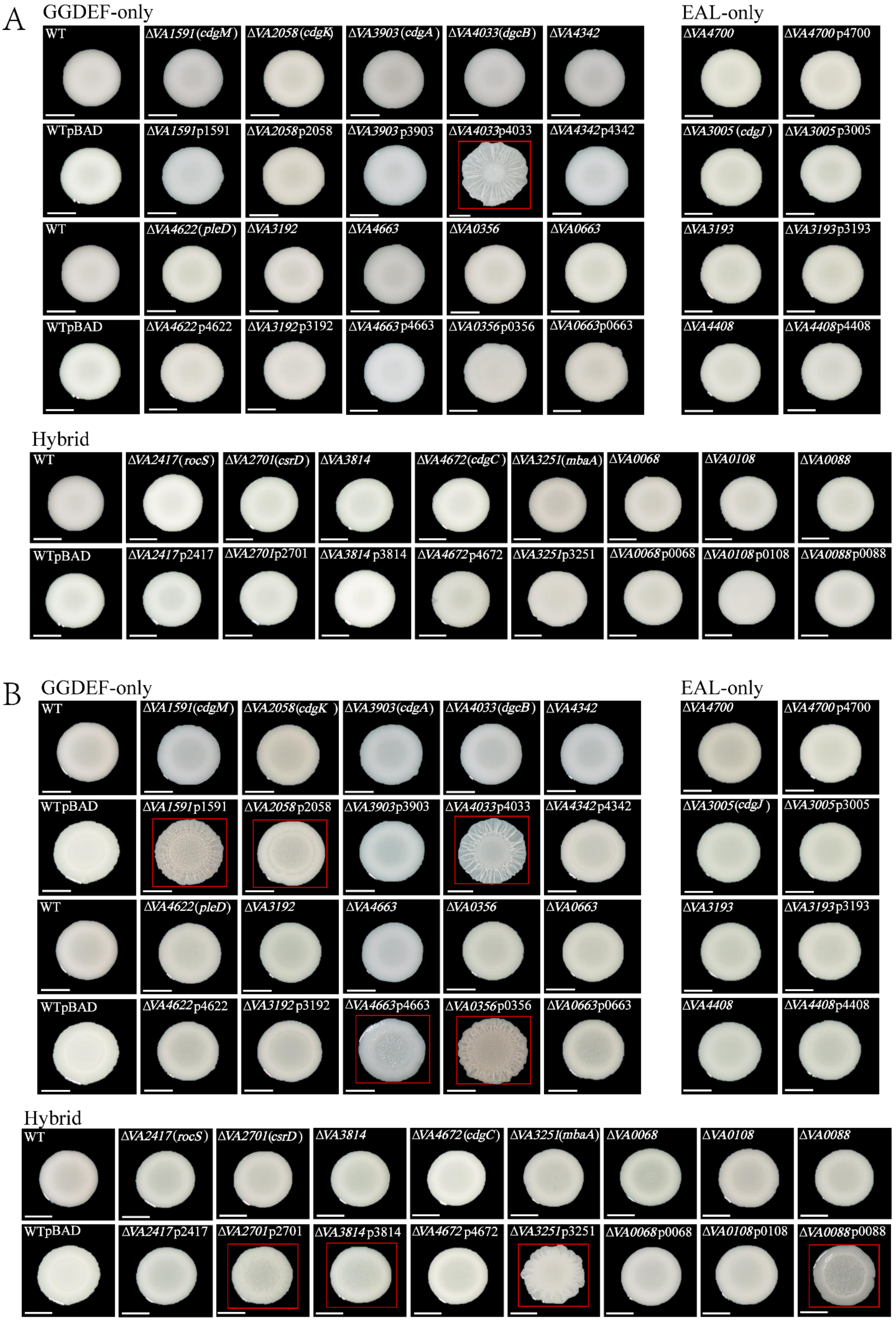
A
GGDEF-only
EAL-only
WT
ΔVA1591(cdgM)
ΔVA2058(cdgK)
ΔVA3903(cdgA)
ΔVA4033(dgcB)
ΔVA4342
ΔVA4700
ΔVA4700p4700
WTpBAD
ΔVA1591p1591
ΔVA2058p2058
ΔVA3903p3903
ΔVA4033p4033
ΔVA4342p4342
ΔVA3005(cdgJ)
ΔVA3005p3005
WT
ΔVA4622(pleD)
ΔVA3192
ΔVA4663
ΔVA0356
ΔVA0663
ΔVA3193
ΔVA3193p3193
WTpBAD
ΔVA4622p4622
ΔVA3192p3192
ΔVA4663p4663
ΔVA0356p0356
ΔVA0663p0663
ΔVA4408
ΔVA4408p4408
Hybrid
WT
ΔVA2417(rocS)
ΔVA2701(csrD)
ΔVA3814
ΔVA4672(cdgC)
ΔVA3251(mbaA)
ΔVA0068
ΔVA0108
ΔVA0088
WTpBAD
ΔVA2417p2417
ΔVA2701p2701
ΔVA3814p3814
ΔVA4672p4672
ΔVA3251p3251
ΔVA0068p0068
ΔVA0108p0108
ΔVA0088p0088
B
GGDEF-only
EAL-only
WT
ΔVA1591(cdgM)
ΔVA2058(cdgK)
ΔVA3903(cdgA)
ΔVA4033(dgcB)
ΔVA4342
ΔVA4700
ΔVA4700p4700
WTpBAD
ΔVA1591p1591
ΔVA2058p2058
ΔVA3903p3903
ΔVA4033p4033
ΔVA4342p4342
ΔVA3005(cdgJ)
ΔVA3005p3005
WT
ΔVA4622(pleD)
ΔVA3192
ΔVA4663
ΔVA0356
ΔVA0663
ΔVA3193
ΔVA3193p3193
WTpBAD
ΔVA4622p4622
ΔVA3192p3192
ΔVA4663p4663
ΔVA0356p0356
ΔVA0663p0663
ΔVA4408
ΔVA4408p4408
Hybrid
WT
ΔVA2417(rocS)
ΔVA2701(csrD)
ΔVA3814
ΔVA4672(cdgC)
ΔVA3251(mbaA)
ΔVA0068
ΔVA0108
ΔVA0088
WTpBAD
ΔVA2417p2417
ΔVA2701p2701
ΔVA3814p3814
ΔVA4672p4672
ΔVA3251p3251
ΔVA0068p0068
ΔVA0108p0108
ΔVA0088p0088

Supplementary Figure S8. Morphological images of colonies of the wild-type strain (or wild-type carrying pBAD vector) and 22 c-di-GMP metabolic gene deletion mutants and corresponding 22 overexpression strains on LBS (A) and LB (B) plates. Experiments were performed in three independent biological replicates and representative images are shown (scaled to equal diameter; bars=5 mm).

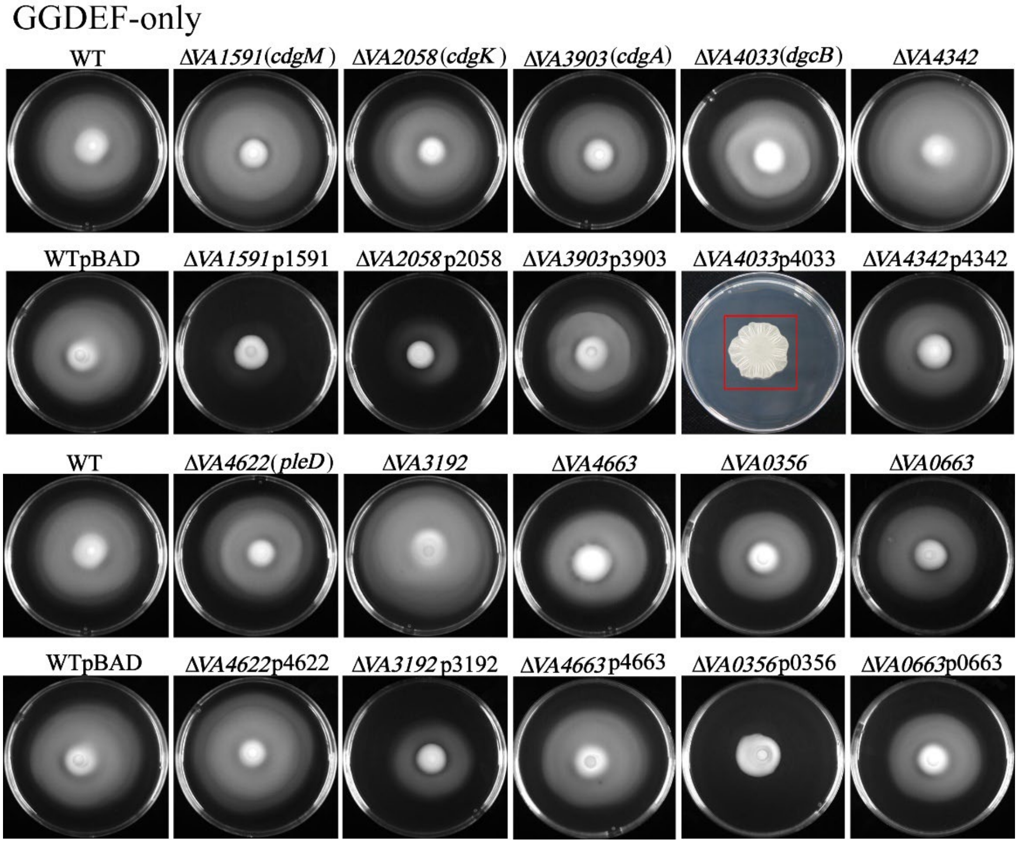

Supplementary Figure S9. Swarming motility assay. Morphological images of colonies of the wild-type strain (or wild-type carrying pBAD vector) and 10 c-di-GMP metabolic gene deletion mutants and corresponding 10 overexpression strains on LBS 0.6% agar plates. Experiments were performed in three independent biological replicates.
